# Association of Ketamine With Psychiatric Symptoms and Implications for Its Therapeutic Use and for Understanding Schizophrenia: A Systematic Review and Meta-analysis

**DOI:** 10.1001/jamanetworkopen.2020.4693

**Published:** 2020-05-21

**Authors:** Katherine Beck, Guy Hindley, Faith Borgan, Cedric Ginestet, Robert McCutcheon, Stefan Brugger, Naomi Driesen, Mohini Ranganathan, Deepak Cyril D’Souza, Matthew Taylor, John H. Krystal, Oliver D. Howes

**Affiliations:** 1 Department of Psychosis Studies, Institute of Psychiatry, Psychology and Neuroscience, King’s College London, London, United Kingdom; 2 Psychiatric Imaging Group, MRC (Medical Research Council) London Institute of Medical Sciences, Hammersmith Hospital, London, United Kingdom; 3 South London and Maudsley NHS (National Health Service) Foundation Trust, London, United Kingdom; 4 Department of Biostatistics and Health Informatics, Institute of Psychiatry, Psychology and Neuroscience, King’s College London, London, United Kingdom; 5 Division of Psychiatry, University College London, London, United Kingdom; 6 Cardiff University Brain Research Imaging Centre, Cardiff, United Kingdom; 7 Yale University Medical School, Veterans Affairs Connecticut Health Care System, West Haven; 8 Department of Psychiatry and National Center for Posttraumatic Stress Disorder (PTSD), Veterans Affairs Connecticut Healthcare System, West Haven; 9 University Department of Psychiatry, Warneford Hospital, Oxford, United Kingdom; 10 Department of Veteran Affairs National Center for Posttraumatic Stress Disorder, Clinical Neurosciences Division, Veterans Affairs Connecticut Healthcare System, West Haven; 11 Institute of Clinical Sciences, Faculty of Medicine, Imperial College London, London, United Kingdom

## Abstract

**Importance:**

Ketamine hydrochloride is increasingly used to treat depression and other psychiatric disorders but can induce schizophrenia-like or psychotomimetic symptoms. Despite this risk, the consistency and magnitude of symptoms induced by ketamine or what factors are associated with these symptoms remain unknown.

**Objective:**

To conduct a meta-analysis of the psychopathological outcomes associated with ketamine in healthy volunteers and patients with schizophrenia and the experimental factors associated with these outcomes.

**Data Sources:**

MEDLINE, Embase, and PsychINFO databases were searched for within-participant, placebo-controlled studies reporting symptoms using the Brief Psychiatric Rating Scale (BPRS) or the Positive and Negative Syndrome Scale (PANSS) in response to an acute ketamine challenge in healthy participants or patients with schizophrenia.

**Study Selection:**

Of 8464 citations retrieved, 36 studies involving healthy participants were included. Inclusion criteria were studies (1) including healthy participants; (2) reporting symptoms occurring in response to acute administration of subanesthetic doses of ketamine (racemic ketamine, s-ketamine, r-ketamine) intravenously; (3) containing a placebo condition with a within-subject, crossover design; (4) measuring total positive or negative symptoms using BPRS or PANSS; and (5) providing data allowing the estimation of the mean difference and deviation between the ketamine and placebo condition.

**Data Extraction and Synthesis:**

Two independent investigators extracted study-level data for a random-effects meta-analysis. Total, positive, and negative BPRS and PANSS scores were extracted. Subgroup analyses were conducted examining the effects of blinding status, ketamine preparation, infusion method, and time between ketamine and placebo conditions. The Meta-analysis of Observational Studies in Epidemiology (MOOSE) and Preferred Reporting Items for Systematic Reviews and Meta-analyses (PRISMA) guidelines were followed.

**Main Outcomes and Measures:**

Standardized mean differences (SMDs) were used as effect sizes for individual studies. Standardized mean differences between ketamine and placebo conditions were calculated for total, positive, and negative BPRS and PANSS scores.

**Results:**

The overall sample included 725 healthy volunteers (mean [SD] age, 28.3 [3.6] years; 533 [73.6%] male) exposed to the ketamine and placebo conditions. Racemic ketamine or S-ketamine was associated with a statistically significant increase in transient psychopathology in healthy participants for total (SMD = 1.50 [95% CI, 1.23-1.77]; *P* < .001), positive (SMD = 1.55 [95% CI, 1.29-1.81]; *P* < .001), and negative (SMD = 1.16 [95% CI, 0.96-1.35]; *P* < .001) symptom ratings relative to the placebo condition. The effect size for this association was significantly greater for positive than negative symptoms of psychosis (estimate, 0.36 [95% CI, 0.12-0.61]; *P* = .004). There was significant inconsistency in outcomes between studies (*I*^2^ range, 77%-83%). Bolus followed by constant infusion increased ketamine’s association with positive symptoms relative to infusion alone (effect size, 1.63 [95% CI, 1.36-1.90] vs 0.84 [95% CI, 0.35-1.33]; *P* = .006). Single-day study design increased ketamine’s ability to generate total symptoms (effect size, 2.29 [95% CI, 1.69-2.89] vs 1.39 [95% CI, 1.12-1.66]; *P* = .007), but age and sex did not moderate outcomes. Insufficient studies were available for meta-analysis of studies in schizophrenia. Of these studies, 2 found a statistically significant increase in symptoms with ketamine administration in total and positive symptoms. Only 1 study found an increase in negative symptom severity with ketamine.

**Conclusions and Relevance:**

This study found that acute ketamine administration was associated with schizophrenia-like or psychotomimetic symptoms with large effect sizes, but there was a greater increase in positive than negative symptoms and when a bolus was used. These findings suggest that bolus doses should be avoided in the therapeutic use of ketamine to minimize the risk of inducing transient positive (psychotic) symptoms.

## Key Points

**Question:**

What psychopathological outcomes are associated with ketamine hydrochloride in healthy volunteers and patients with schizophrenia, and what factors are associated with these outcomes?

**Findings:**

This meta-analysis of 36 studies, including 725 unique healthy participants, found that the acute administration of ketamine relative to placebo was associated with a meaningful increase in positive and negative symptoms of psychosis in healthy volunteers and patients with schizophrenia. This association was greater for positive compared with negative symptoms and when a bolus was given with the infusion relative to an infusion alone.

**Meaning:**

These findings suggest that ketamine is associated with psychosis-like symptoms in healthy volunteers and that the bolus administration of ketamine should be avoided when it is used in therapeutic contexts.

## Teaser

This meta-analysis assesses the psychopathological outcomes in healthy volunteers and patients with schizophrenia treated with ketamine and the experimental factors associated with these findings.

## Introduction

Ketamine hydrochloride was first synthesized in 1962.^1^ It is a phencyclidine derivative that acts on the glutamate system by antagonizing *N*-methyl-D-aspartate (NMDA) receptors.^1^ Ketamine has been used to model the symptoms of schizophrenia and is used in the treatment of severe depression and pain management^2^ as well as being used recreationally. Misuse can be hazardous, leading to drug addiction.

In the 1960s, NMDA antagonists, such as ketamine, were identified as inducing clinical symptoms similar to those seen in schizophrenia, more so than other psychotomimetics used in past drug models of psychosis.^3,4^ In particular, in addition to inducing positive symptoms, such as perceptual changes and delusions, ketamine induces negative symptoms, such as blunted affect and emotional withdrawal.^5^ Many studies have been conducted to investigate its effect on healthy people, but the methods vary greatly, and the observed behavioral responses differ.

Despite the recognition that ketamine can induce transient schizophrenia-like symptoms,^5^ the consistency and magnitude of its effect on positive and negative symptoms remains unclear. Moreover, it is unclear how blinding status, ketamine preparation, infusion method, and time between the ketamine and placebo conditions are associated with the generation of symptoms.

The development of ketamine and its derivatives as antidepressants^6,7^ means that determining the extent to which ketamine induces schizophrenia-like or psychotomimetic symptoms and what factors are associated with this outcome is particularly timely in order to understand and minimize the risks of adverse events associated with the therapeutic use of ketamine. We also aimed to evaluate outcomes in patients with schizophrenia to determine whether they are more sensitive to ketamine.

We therefore conducted a systematic review and meta-analysis of the association of ketamine with positive, negative, and total psychopathological outcomes in healthy volunteers and patients with schizophrenia. Many studies use ketamine to inform understanding of the mechanisms underlying schizophrenia. This specific use of ketamine is the main focus of our review, but we also use the findings to inform understanding of other uses of ketamine.

## Methods

### Selection Procedures

A meta-analysis was performed according to the Meta-analysis of Observational Studies in Epidemiology (MOOSE)^8^ and Preferred Reporting Items for Systematic Reviews and Meta-analyses (PRISMA)^9^ frameworks. Three authors (K.B., G.H., and F.B.) independently searched MEDLINE (from 1946 to February 3, 2020), Embase (from 1974 to February 3, 2020), and PsychINFO (from 1806 to January 27, 2020). The following keywords were used: (*Ketamine*) and (psycho* NOT psychotherapy or schiz* or *BPRS* or *brief psychiatric rating scale* or *PANSS* or *positive and negative syndrome scale* or positive symp* or negative symp*). Meta-analyses and systematic and narrative review articles were hand-searched for additional reports. Abstracts were screened, and the full texts of suitable studies were obtained. If full texts were not available, authors were contacted and full content was requested. Authors were also contacted when Brief Psychiatric Rating Scale (BPRS) or Positive and Negative Syndrome Scale (PANSS) subscales (total, negative, or positive) were missing or if the individual items included in the positive or negative scales were not reported. Three authors (K.B., G.H., and F.B.) selected the final studies included in the meta-analysis based on the following criteria.

### Selection Criteria for the Meta-analysis of Ketamine’s Effects in Healthy Volunteers

Inclusion criteria were studies (1) including healthy participants, (2) reporting symptoms occurring in response to acute administration of subanesthetic doses of ketamine (racemic ketamine, s-ketamine, or r-ketamine) intravenously, (3) containing a placebo condition with a within-participant, crossover design, (4) measuring total positive or negative symptoms using the BPRS or PANSS, and (5) providing data allowing the estimation of the mean difference and deviation between the ketamine and placebo condition. We used the PANSS and BPRS scales as the measures of symptom severity because they are well validated, standardized assessments of psychopathology used in both healthy participants and patients with schizophrenia.^10,11^ They assess the same symptom dimensions and are commonly combined in meta-analyses.^12^ We included all versions of the total BPRS because often the version was not specified. All versions measure the same rating items, but some include more items than others. However, all included studies are within-person studies, and so this should not affect the analysis. Exclusion criteria consisted of 1 or more of the following factors: (1) no placebo condition, (2) no report of any total, negative, or positive scores (see the following sections for more details), (3) absence of measures in either the ketamine or the placebo condition, (4) no report of original data, (5) no data provided that enabled the standardized mean differences (SMDs) to be calculated (such as the SD or the standard error of the mean), (6) no more than 2 participants in each group, and/or (7) concurrent administration of other pharmacological compounds in addition to ketamine.

### Selection Criteria for the Meta-analysis of Ketamine’s Effects in Schizophrenia

The selection criteria for studies investigating the effect of ketamine in patients with schizophrenia were the same as the criteria for healthy volunteers. The only additional criterion was for participants to have a *Diagnostic and Statistical Manual of Mental Disorders* or *International Statistical Classification of Diseases and Related Health Problems, Tenth Revision* diagnosis of schizophrenia or schizoaffective disorder.

### Additional Symptom Subdomain Inclusion Criteria for Both Meta-analyses

Studies used different combinations of symptom items in their positive and negative BPRS scores. We included studies in the negative analysis if their BPRS scale included all 3 negative symptom items: blunted affect, emotional withdrawal, and motor retardation. We included studies in the positive analysis if they included more than 3 positive symptom items: conceptual disorganization, hallucinatory behavior, unusual thought content, and suspiciousness. These symptom items correlate most strongly and reliably with validated scales of positive and negative symptoms^13^: the Scale for the Assessment of Positive Symptoms^14^ and Scale for the Assessment of Negative Symptoms,^15^ respectively. If the symptom items included in the scale were not documented, we requested the information from authors (eMethods 1 in the Supplement).

### Recorded Variables

The primary outcome measures were the effect sizes for total, positive, and negative BPRS and PANSS scores in healthy participants or in patients with schizophrenia for ketamine compared with placebo conditions. Data were extracted from every study for author, year of publication, number of participants, participant age, sex, diagnosis, study design, details of the placebo condition, past or present psychiatric diagnoses among healthy volunteers, recent substance misuse or dependence history, family history of psychosis, major medical or neurological disorder, prior exposure to ketamine, concurrent psychotropic medication use, ketamine preparation, dose and timing of ketamine administration relative to the symptom measures, and mean (SD) measure of symptoms in the ketamine and placebo conditions. Plot digitizer software was used to examine reliability for the data from studies in which data were only available in a plot format.

The highest available ketamine dose was selected if multiple doses were reported. All data sets included in the meta-analysis were independent, and there was no overlap in the participants included in the meta-analyses. The raw data are provided in eTables 1 to 4 in the Supplement.

### Risk of Bias

Risk of bias was assessed using the Newcastle-Ottawa tool for assessing risk in nonrandomized studies and the Cochrane assessment of risk of bias tool.^16,17^ Scores were calculated by 2 investigators (K.B., G.H.). Studies with scores of at least 7 were considered to have a low risk of bias (eMethods 2-4 in the Supplement).

### Statistical Analysis

Statistical analyses were conducted using the metafor package, version 1.9-9, with R software, version 3.3.1 (R Project for Statistical Computing). Random-effects models based on restricted maximum likelihood estimation were used in all analyses. Random-effects models were deemed preferable for this analysis owing to substantial between-study differences in study design. Effect sizes or SMDs for individual studies were estimated by calculating the standardized mean change scores. Mean differences in symptom measurements between the ketamine and placebo conditions were used to calculate the standardized mean change score. The 95% CI of the effect size was also calculated.

The SMD was defined for each study as follows^18^:


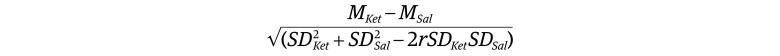


where *M_Ket_* and *M_Sal_* are the mean scores and SD*_Ket_* and SD*_Sal_* are the SDs for the ketamine and saline (placebo) conditions, respectively, with *r* denoting the between-condition correlation for symptom scores under saline and ketamine conditions. The correlation coefficient was set to 0.5 for all studies in our main analysis based on evidence from studies in schizophrenia.^19,20^ However, a sensitivity analysis was performed to evaluate the influence of this assumption on our main results by refitting our model using *r* values ranging from 0.1 and 0.7 (eMethods 5 and 6 in the Supplement).

To determine whether ketamine had a greater association with positive or negative symptoms, a multivariate meta-analytic approach was adopted using an unstructured variance-covariance matrix. Because within-study correlations between positive and negative symptom scores are not reported, we estimated the correlation coefficient to be 0.5 based on prior studies.^20^ To investigate the influence of this value on the findings, we conducted sensitivity analyses using correlation coefficients of 0.1 and 0.7 (eMethods 7 and 8 in the Supplement).^21^

Inconsistency or heterogeneity across studies was assessed using the Cochran Q statistic^22^ and *I*^2^ statistic.^23^ An *I*^2^ statistic of less than 25% was taken to indicate low inconsistency; 25% to 75%, medium inconsistency; and greater than 75%, high inconsistency. The *I*^2^ statistics were calculated for each subgroup analysis. Leave-one-out sensitivity analyses were also conducted.

Publication bias and selective reporting were assessed using the Egger regression test of the intercept^24^ and were represented diagrammatically with funnel plots as recommended by the Cochrane Collaboration (eFigures 1-3 in the Supplement). Trim-and-fill analyses were also conducted.

Secondary subgroup and meta-regression analyses were performed to examine the effects of study design. Specifically, we compared effect size in double-blind vs single-blind or unblinded studies; s-ketamine vs racemic ketamine; bolus followed by constant infusion administration vs infusion alone; and single-day (ketamine and placebo were given on the same day) vs multiple-day (ketamine and placebo given on different days) studies. In addition, we compared the effect size from studies using the BPRS with those using the PANSS to determine whether the method of measuring symptoms was associated with the magnitude of the effect. The statistical significance of subgroup differences was determined by fitting separate random-effects models for each subgroup and then comparing subgroup summary estimates in a fixed-effects model with a Wald-type test. A significance level of *P* < .05 (2 tailed) was adopted (see eMethods 9 and 10 in the Supplement for further details).

## Results

### Retrieved Studies for the Meta-analysis of Healthy Volunteers

**Figure 1.**
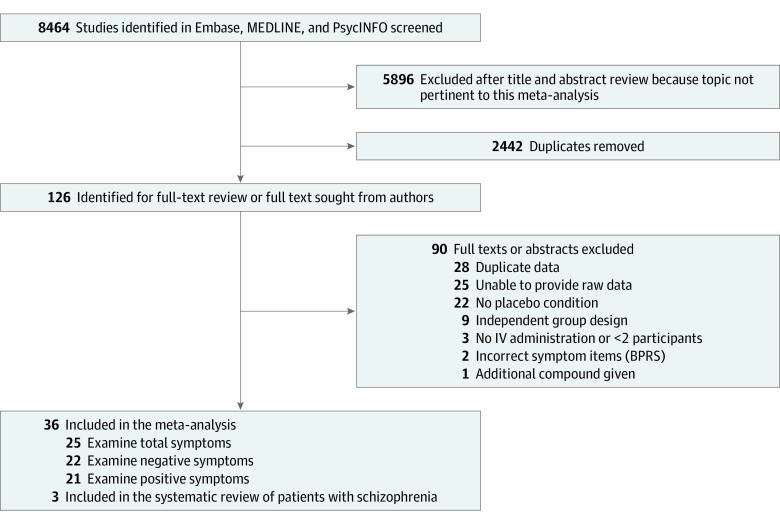
Search Process Summarizing the Review and Exclusion of Studies BPRS indicates Brief Psychiatric Rating Scale; IV, intravenous.

A total of 36 studies involving 725 unique participants (mean [SD] age, 28.3 [3.6] years; 533 male [73.6%] and 192 female [26.5%]) were included in the meta-analysis.^3,25,28,29,30,31,32,33,34,35,36,37,38,39,40,41,42,43,44,45,46,47,48,49,50,51,52,53,54,55,56,57,58,59,60,61^ Figure 1 shows the PRISMA flowchart. The included studies are summarized in the Table, with further details in eTable 5 in the Supplement. Ketamine was administered intravenously in all studies. The search identified 2 additional studies using inhaled administration, but these did not have data available.^62,63^

**Table.** Summary of Sample and Study Characteristics of Included Studies Involving Healthy Volunteers and Patients With Schizophrenia^a^

Source	Sample size, No.	Age, mean (SD), y	Sex, No. male:female	Blinded	Randomized	Placebo condition	Symptom subscales reported	Length of ketamine infusion before symptom assessment, min
**BPRS Studies**								
Kraguljac et al,^30^ 2017	15	24.8 (3.49)	10:5	No	No	Saline	Total, positive (2), and negative	NR
Kort et al,^43^ 2017	31	27.0 (4.3)	19:12	Double	Yes	Saline	Total	NR
Duncan et al,^36^ 2001	16	33.3 (3.1)	16:0	Double	Yes	Saline	Total and negative	50
Parwani et al,^37^ 2005	13	31.9 (9.6)	5:8	Double	Yes	Saline	Total	15
Rowland et al,^38^ 2005	10	24.7 (3.4)	10:0	Double	Yes	Saline	Total	45
Abel et al,^42^ 2003	8	28.75	8:0	Double	Yes	Saline	Total	15
Anand et al,^44^ 2000	16	34.0 (12.0)	8:8	Double	Yes	Saline	Positive (1) and negative	5
Krystal et al,^35^ 1998	23	30.0	19:11	Double	Yes	Saline	Positive (1) and negative	60 for both subscales
Breier et al,^39^ 1997	17	30.4 (6.8)	15:2	Double	Yes	Saline	Positive (1)	NR
van Berckel et al,^40^ 1998	18	23.7 (2.4)	18:0	Double	Yes	NR	Total	40
Malhotra et al,^25^ 1997	16	27.8 (1.9)	12:4	Double	Yes	Saline	Total and negative	55
Krystal et al,^45^ 1999	20	28	10:10	Double	Yes	Saline	Positive (1) and negative	60 for both subscales
Krystal et al,^46^ 2003	26	29.1 (9)	19:7	Double	Yes	Saline	Positive (1) and negative	80
Micallef et al,^47^ 2002	8	27.0	4:4	Double	Yes	Saline	Positive (1) and negative	NR
Rowland et al,^52^ 2010	9	30.8	4:5	Double	Yes	Saline	Total	NR
Newcomer et al,^31^ 1999	15	21.7 (3.2)	15:0	Double	Yes	Saline	Total and positive (1)	30
Stone et al,^53^ 2011	8	28 (5.9)	8:0	Double	Yes	Saline	Total	NR
Boeijinga et al,^48^ 2007	12	39.6 (4.8)	12:0	Double	Yes	Saline	Total	30
Abdallah et al,^54^ 2018	14	NR	NR	Single	No	Saline	Total and negative	120
Passie et al,^55^ 2003	12	26.8 (3.31)	12:0	Double	Yes	Saline	Total	NR
Horacek et al,^56^ 2010	20	29.9 (5.69)	13:7	Double	Yes	Saline	Total	NR
Morgan et al,^57^ 2011	16	22.4	10:8	Double	No	Saline	Total	NR
**PANSS Studies**								
Thiebes et al,^29^ 2017	24	25 (2.64)	24:0	Single	Yes	Saline	Total, positive, negative, and factor scores	NR
Powers et al,^58^ 2015	19	27.5	10:10	No	No	Saline	Positive and negative	NR
Höflich et al,^49^ 2015	30	25 (4.58)	NR	Double	Yes	Saline	Total, positive, and negative	NR
Nagels et al,^59^ 2011	15	27 (3.6)	15:0	Double	Yes	Saline	Total, positive, and negative	NR
Driesen et al,^60^ 2013	22	29.14 (7.07)	14:8	No	No	Saline	Positive and negative	45
Vernaleken et al,^50^ 2013	10	24.4 (3.9)	10:0	Single	Yes	Saline	Total, positive, and negative	NR
Krystal et al,^3^ 2005	27	30.96	16:11	Double	Yes	Saline	Positive, negative, and factor score	60 for both subscales
Krystal et al,^32^ 2006	31	28.1 (7.6)	NR	Double	Yes	Saline	Total, positive, negative, and factor score	NR
Kleinloog et al,^28^ 2015	30	NR	15:15	Double	Yes	Saline	Positive and negative	NR
D’Souza et al,^51^ 2012	32	27 (8.42)	NR	Double	Yes	Saline	Total, positive, and negative	NR
Grent-‘t-Jong et al,^33^ 2018	14	29 (0.9)	12:2	Single	Yes	Saline	Total, negative, and positive	NR
D’Souza et al,^34^ 2018	26	29.8 (9.56)	21:5	NR	No	Saline	Negative and positive	NR
Mathalon et al,^61^ 2014	9	29.8 (7.9)	5:4	Double	Yes	Saline	Total	1
Dickerson et al,^41^ 2010	93	24.29 (2.62)	47:46	Single	Yes	Saline	Total, positive, negative, and factor score	45
**Schizophrenia (BPRS)**								
Lahti et al,^26^ 2001	17	31.6 (7.8)	11:6	Double	Yes	NR	Total, positive (2), and negative	20
Malhotra et al,^25^ 1997	13	31.3 (2.8)	10:3	Double	Yes	Saline	Total	55
Malhotra et al,^27^ 1998	18	34.7 (2.3)	13:5	Double	Yes	NR	Positive (1) and negative	35

Abbreviations: BPRS, Brief Psychiatric Rating Scale; NR, not reported; PANSS, Positive and Negative Syndrome Scale.

^a^ The BPRS measure includes the following positive symptoms (1): conceptual disorganization, suspiciousness, hallucinatory behavior, and unusual thought content; positive symptoms (2): conceptual disorganization, hallucinatory behavior, and unusual thought content; and negative symptoms: blunted affect, emotional withdrawal, and motor retardation. Further details including doses administered are reported in eTables 5 and 6 in the Supplement.

### Total Psychopathological Symptoms

**Figure 2.**
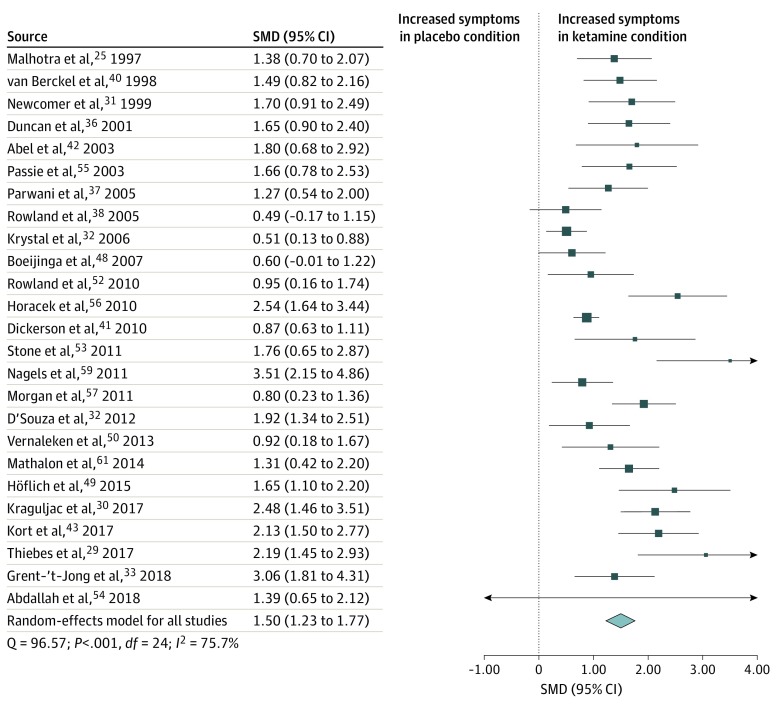
Standardized Mean Difference (SMD) in Total Symptoms Scores for Healthy Volunteers After Ketamine vs Placebo Administration Scores include Positive and Negative Syndrome Scale and Brief Psychiatric Rating Scale. A statistically significant increase in total symptoms occurred in healthy volunteers in the ketamine condition compared with the placebo condition (SMD = 1.50 [95% CI, 1.23-1.77]; *P* < .001). Each square shows the effect size for a single study, with the horizontal error bars representing the width of the 95% CI. The size of the squares reflects the weight attributed to each study. The diamond illustrates the summary effect size, and the width of the diamond depicts the width of the overall 95% CI.

Total symptom scores were analyzed using data from 25 studies^25,29,30,31,32,33,36,37,38,40,41,42,43,48,49,50,51,52,53,54,55,56,57,59,61^ including 491 healthy participants exposed to the ketamine and placebo conditions. Total symptom scores were increased in the ketamine condition compared with the placebo condition (SMD = 1.50 [95% CI, 1.23-1.77]; *P* < .001) (Figure 2). The finding remained statistically significant in all iterations of the leave-one-out analysis (SMD range, 1.44-1.55; *P* < .001).

Statistically significant between-sample inconsistency was found, with an *I*^2^ value of 75.7% (Cochran Q = 96.57; *P* < .001). The Egger test (*z* = 4.27; *P* < .001) suggested that publication bias was statistically significant. Trim-and-fill analysis estimated 3 missing studies on the left side of eFigure 1 in the Supplement, indicating that negative studies may have not been reported. However, our results remained statistically significant when the putative missing studies were included (SMD = 1.37 [95% CI, 1.07-1.67]; *P* < .001). Meta-regressions of effect sizes against age (n = 24)^5,29,30,31,32,33,36,37,38,40,41,42,43,48,49,50,51,52,53,55,56,57,59,61^ and sex (n = 22)^25,29,30,31,33,36,37,38,40,41,42,43,48,50,51,52,53,55,56,57,59,61^ showed that neither factor was a statistically significant moderator of effect sizes.

#### Ketamine Preparation

Both racemic ketamine and s-ketamine preparations resulted in a statistically significant increase in total symptom scores compared with placebo. Large effect sizes were found for racemic ketamine (SMD = 1.40 [95% CI, 1.12-1.68]; *P* < .001) and s-ketamine (SMD, 2.03 [95% CI, 1.15-2.92]; *P* < .001). There was no significant difference between the methods on the magnitude of the effect size.

#### Blinding Method

Unblinded or single-blind methods (SMD = 1.71 [95% CI, 1.02-2.39]; *P* < .001) and double-blind methods (SMD = 1.45 [95% CI, 1.15-1.75]; *P* < .001) both resulted in a statistically significant association of the ketamine condition with total symptoms. There was no significant difference between the methods on the magnitude of the effect size.

#### Infusion Method

Bolus and a continuous infusion (SMD = 1.55 [95% CI, 1.23-1.88]; *P* < .001) and a continuous infusion only (SMD = 1.27 [95% CI, 0.73-1.81]; *P* < .001) were both associated with a statistically significant increase in total symptoms. There was no significant difference between the methods on the magnitude of the effect size.

#### Single-Day vs Multiple-Day Studies

Two single-day studies^29,30^ (SMD = 2.29 [95% CI, 1.69-2.89]; *P* < .001) and 17 multiple-day studies^25,31,36,37,38,40,41,42,43,48,50,51,52,53,55,56,61^ (SMD = 1.39 [95% CI, 1.12-1.66]; *P* < .001) were associated with a statistically significant increase in total symptoms. Studies in which ketamine and placebo conditions were conducted on the same day found a significantly greater magnitude of effect (effect size, 2.29 [95% CI, 1.69-2.89] vs 1.39 [95% CI, 1.12-1.66]; *P* = .007) (eFigure 4 in the Supplement).

### Positive Psychotic Symptoms

**Figure 3.**
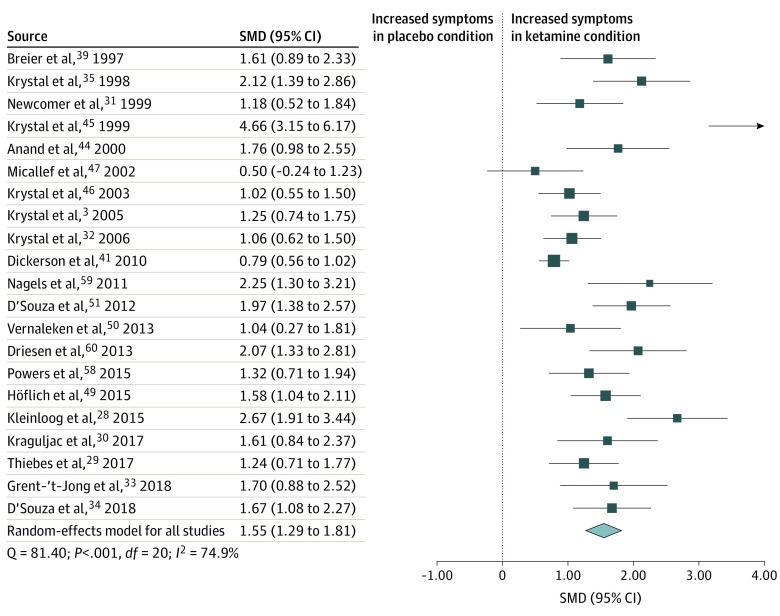
Standardized Mean Difference (SMD) in Positive Symptom Scores for Healthy Volunteers After Ketamine vs Placebo Administration Scores include Positive and Negative Syndrome Scale and Brief Psychiatric Rating Scale. A statistically significant increase in positive symptoms occurred in healthy volunteers in the ketamine condition compared with the placebo condition (SMD = 1.55 [95% CI, 1.29-1.81]; *P* < .001). Each square shows the effect size for a single study, with the horizontal error bars representing the width of the 95% CI. The size of the squares reflects the weight attributed to each study. The diamond illustrates the summary effect size, and the width of the diamond depicts the width of the overall 95% CI.

Positive symptom scores were analyzed using data from 21 studies^3,28,29,30,31,32,33,34,35,39,41,44,45,46,47,49,50,51,58,59,60^ consisting of 513 healthy participants exposed to the ketamine and placebo conditions. Positive symptom scores were transiently increased in the ketamine condition compared with the placebo condition (SMD = 1.55 [95% CI, 1.29-1.81]; *P* < .001) (Figure 3). The result remained statistically significant in all iterations of the leave-one-out analysis (SMD range, 1.47-1.60; *P* < .001).

Statistically significant between-sample inconsistency was found, with an *I*^2^ value of 74.9% (Cochran Q = 81.40; *P* < .001). Findings of the Egger test (*z* = 5.06; *P* < .001) suggested that publication bias was significant. Trim-and-fill analysis estimated 1 missing study on the left side (eFigure 2 in the Supplement). Results remained statistically significant with putative missing studies included (SMD = 1.49 [95% CI, 1.18-1.80]; *P* < .001). Meta-regressions of effect sizes against age (n = 20)^3,29,30,31,32,33,34,35,39,41,44,45,46,47,49,50,51,58,59,60^ or sex (n = 19)^3,28,29,30,31,33,34,35,39,41,44,45,46,47,50,51,58,59,60^ showed that neither was a statistically significant moderator of effect sizes.

#### Ketamine Preparation

Both racemic ketamine (SMD = 1.50 [95% CI, 1.17-1.82]; *P* < .001) and s-ketamine (SMD = 1.70 [95% CI, 1.23-2.18]; *P* < .001) preparations resulted in a statistically significant increase in positive symptom scores compared with placebo, both with large effect sizes. There was no significant difference between the methods on the magnitude of the effect size.

#### Blinding Method

Unblinded or single-blind (SMD = 1.32 [95% CI, 0.96-1.67]; *P* < .001) and double-blind (SMD = 1.68 [95% CI, 1.30-2.07]; *P* < .001) methods resulted in a statistically significant effect of ketamine condition on the positive symptoms. However, there was no significant difference in the magnitude of the effect between the 2 methods.

#### Infusion Method

Both a bolus followed by continuous infusion method (n = 19)^28,29,30,31,32,33,34,35,39,41,44,45,49,50,51,58,59,60,64^ (SMD = 1.63 [95% CI, 1.36-1.90]; *P* < .001) and a continuous infusion alone (n = 2)^46,47^ (SMD = 0.84 [95% CI, 0.35-1.33]; *P* < .008) induced a statistically significant increase in positive symptoms. However, studies using a bolus and continuous infusion method induced a statistically significantly greater magnitude of effect (effect size, 1.63 [95% CI, 1.36-1.90) compared to continuous infusion alone (effect size, 0.84 [95% CI, 0.35-1.33]; *P* = .006) (eFigure 5 in the Supplement).

#### Single-Day vs Multiple-Day Studies

Single-day (SMD = 1.54 [95% CI, 1.19-1.89]; *P* < .001) and multiple-day (SMD = 1.53 [95% CI, 1.15-1.90]; *P* < .001) studies both resulted in a statistically significant increase in positive symptoms. There was no significant difference in the magnitude of the effect between the 2 methods.

### Negative Symptoms

**Figure 4.**
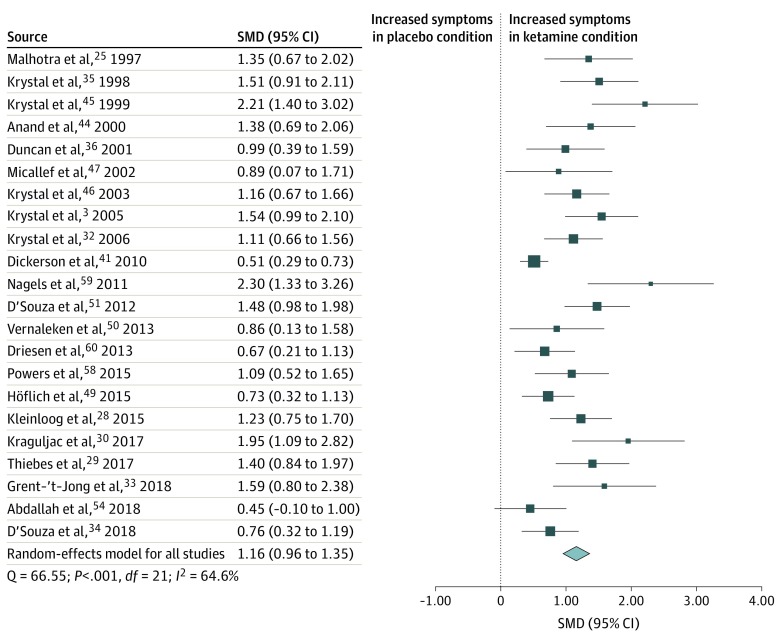
Standardized Mean Difference (SMD) in Negative Symptom Scores in Healthy Volunteers After Ketamine vs Placebo Administration Scores include Positive and Negative Syndrome Scale and Brief Psychiatric Rating Scale. A statistically significant increase in negative symptoms occurred in healthy volunteers in the ketamine condition compared with the placebo condition (SMD = 1.16 [95% CI, 0.96-1.35]; *P* < .001). Each square shows the effect size for a single study, with the horizontal error bars representing the width of the 95% CI. The size of the squares reflects the weight attributed to each study. The diamond illustrates the summary effect size, and the width of the diamond depicts the width of the overall 95% CI.

Negative symptom scores were analyzed using data from 22 studies^3,25,28,29,30,32,33,34,35,36,41,44,45,46,47,49,50,51,54,58,59,60^ consisting of 527 healthy participants exposed to the ketamine and placebo conditions. Negative symptom scores were transiently increased in the ketamine condition compared with the placebo condition (SMD = 1.16 [95% CI, 0.96-1.35]; *P* < .001) (Figure 4). The result remained statistically significant in all iterations of the leave-one-out analysis (SMD range, 1.11-1.19; *P* < .001).

Statistically significant between-sample inconsistency was found, with an *I*^2^ value of 64.6% (Cochran Q = 66.55; *P* < .001). Findings of the Egger test (*z* = 5.12; *P* < .001) suggested that publication bias was significant. Trim-fill analysis estimated 2 missing studies on the left side of eFigure 3 in the Supplement. Results remained statistically significant with putative missing studies included (SMD = 1.09 [95% CI, 0.89-1.30]; *P* < .001). Meta-regressions of effect sizes against age (n = 20)^3,25,29,30,32,33,34,35,36,41,44,45,46,47,49,50,51,58,59,60^ or sex (n = 19)^3,25,28,29,30,33,34,35,36,41,44,45,46,47,50,51,58,59,60^ showed that neither was a statistically significant moderator of effect sizes.

#### Ketamine Preparation

Both racemic ketamine (SMD = 1.13 [95% CI, 0.90-1.36]; *P* < .001) and s-ketamine (SMD = 1.25 [95% CI, 0.86-1.64]; *P* < .001) preparations resulted in a statistically significant transient increase in negative symptom scores compared with placebo, with large effect sizes. There was no significant difference between the methods on the magnitude of the effect size.

#### Blinding Method

Unblinded or single-blind (SMD = 0.98 [95% CI, 0.63-1.34]; *P* < .001) and double-blind (SMD = 1.29 [95% CI, 1.09-1.50]; *P* < .001) methods resulted in a statistically significant association of the ketamine condition with negative symptoms. There was no significant difference between the methods on the magnitude of the effect size.

#### Infusion Method

Both bolus and a continuous infusion (SMD = 1.19 [95% CI, 0.96-1.41]; *P* < .001) and a continuous infusion only (SMD = 1.06 [95% CI, 0.71-1.40]; *P* < .001) were associated with a statistically significant increase in negative symptoms. There was no significant difference between the methods on the magnitude of the effect size.

#### Single-Day vs Multiple-Day Studies

Single-day (SMD = 1.01 [95% CI, 0.56-1.47]; *P* < .001) and multiple-day (SMD = 1.16 [95% CI, 0.94-1.39]; *P* < .001) studies both resulted in a statistically significant increase in negative symptoms. However, there was no significant difference in the magnitude of the effect between the 2 methods.

### Comparison of Positive and Negative Effect Sizes

A comparison of effect sizes demonstrated that the ketamine condition had a greater association with positive symptoms compared with negative symptoms (estimate, 0.36 [95% CI, 0.12-0.61]; *z* = 2.90; *P* = .004).

Subanalyses of BPRS and PANSS scales are presented in eMethods 10 in the Supplement. In summary, there was no significant difference between the 2 measures for any of the symptom domains (total, positive, and negative). Inconsistency analyses for the subanalyses are presented in eMethods 9 in the Supplement.

### Effects of Ketamine in Patients With Schizophrenia

After 7 studies with overlapping data sets were excluded,^64,65,66,67,68,69,70^ 3 studies were included in the analysis of patients with schizophrenia.^25,26,27^ No meta-analysis was possible because there were an insufficient number of papers. Studies with change scores were included in this section of the review.

Two studies^25,26^ examined the association of acute ketamine administration on total BPRS scores in patients with schizophrenia, and both found that ketamine was associated with a statistically significant increase in total BPRS scores. Two studies^26,27^ investigated the association of ketamine administration with positive and negative BPRS scores in patients with schizophrenia. Both found ketamine was associated with a statistically significant transient increase in positive symptoms. One study^27^ found ketamine was associated with a statistically significant increase in negative symptoms, but the other study^26^ to assess this factor did not find a significant association of ketamine with negative symptoms. The findings of these studies are summarized in eTable 4 in the Supplement.

### Risk of Bias Across Studies

Eight studies^29,41,50,51,54,55,57,58^ had a high risk of bias when the Newcastle-Ottawa tool was used to assess bias, mainly owing to not documenting certain aspects of the design protocol and therefore losing a point for being unclear. The Cochrane tool for assessment of bias across studies highlighted an unclear risk of bias across the selection bias domain but low risk across all other domains (performance bias, detection bias, attrition bias, and reporting bias) (eMethods 2-4 in the Supplement).

## Discussion

Our main findings were that acute ketamine administration was associated with a large effect size for increases in positive, negative, and total symptom scores in healthy volunteers. Moreover, ketamine was associated with greater increases in positive symptoms than in negative symptoms.

Insufficient studies were available to conduct a meta-analysis of the association of ketamine with psychopathology in schizophrenia. Although transient increases in positive, negative, and total symptoms in patients with schizophrenia were reported, given the limited data, firm conclusions on effects in schizophrenia cannot be drawn, and further studies are needed. These findings extend the understanding of the symptoms associated with ketamine by showing that either racemic ketamine or s-ketamine are associated with positive, negative, and total symptoms in healthy volunteers with very large effect sizes across study settings and designs. To give some clinical context to the increased effect sizes seen with ketamine administration, the average mean difference in the total PANSS scores between the placebo and ketamine conditions was 18.40. Were this increase in symptom rating to occur in a patient with schizophrenia, it would approximately equate to a change from mild illness severity to markedly ill on the Clinical Global Impression Scale and represent a clinically meaningful increase in symptoms.^71^

This study identifies high levels of between-study inconsistency. Our subgroup analyses indicate that this inconsistency could be owing to study design factors. Specifically, studies that used a bolus plus infusion protocol showed larger increases (approximately double) in positive symptoms than those using only a continuous infusion. Moreover, studies that administered ketamine and placebo on the same day found a greater increase in total symptoms. The first finding could be owing to a faster time to and/or higher peak concentration of ketamine, consistent with a study showing a positive association between ketamine concentration and symptom induction.^28^ It is less clear why giving ketamine and placebo on the same day was associated with greater induction of symptoms, but this factor could reflect unblinding because one study was unblinded^29^ and the other was single blinded with the condition order randomized.^30^ Another explanation might be that both conditions on the same day controls better for the day-to-day variance that may occur in mood and biology. When heterogeneity was assessed for each individual subgroup, it was moderate to high for most analyses, suggesting that these subgroups did not account for all of the inconsistency seen within the meta-analysis.

### Association of Age and Sex With Ketamine-Induced Psychopathology

Neither age nor sex were associated with the severity of psychotic symptoms induced by ketamine in healthy volunteers. However, the studies included in our meta-analysis only include adults (range of mean ages, 22-40 years). In children, fewer ketamine-induced symptoms might occur because children are less likely to experience psychotic symptoms than adults when given ketamine for anesthesia.^1^ However, animal studies find that ketamine has a greater neurotoxic effect in the period from puberty to early adulthood.^72^ Sex did not moderate the magnitude of effect for any of the symptom measures in our study, consistent with findings by Morgan and colleagues^73^ but in contrast with preclinical evidence that female rats are more susceptible than male rats to the neurotoxic^74^ and behavioral^75^ effects of ketamine. This difference between clinical and preclinical evidence may reflect the higher doses used in the animal studies (5-180 mg/kg) compared with humans (approximately 0.65 mg/kg).

### Implications for Future Study Design and Reporting

Our findings are of particular relevance for the therapeutic use of ketamine and for future study design. First, we provide evidence that the use of bolus plus continuous infusion is associated with larger transient psychotomimetic effects. Second, inadequate reporting of methods precluded our ability to test the effects of other key methodological factors. One recommendation from our findings is therefore for future studies to report methods with greater detail to enable these factors to be investigated and aid replication.^76^ Details of specific relevance to studies such as these include the dose of ketamine and fasting status before receiving ketamine.

### Ketamine Model of Schizophrenia

We found that ketamine was associated with the induction of both transient positive and negative symptoms of schizophrenia in healthy people and with worsened symptoms in patients with schizophrenia. To the extent that any drug can model a complex disorder such as schizophrenia, the results of this meta-analysis support the use of ketamine to model schizophrenia-like or psychotomimetic symptoms and suggest that it provides a more comprehensive model of schizophrenia than drugs such as amphetamine, which does not reliably induce negative symptoms.^3^ However, we found that the induction of negative symptoms is statistically significantly less marked than that of positive psychotic symptoms in healthy people, and it was only seen in 1 of the 2 studies in schizophrenia.^27^ The negative symptom analysis had an extra study with a continuous infusion method. This difference may have reduced the effect because the continuous infusion method appears less likely than the bolus and continuous method to induce psychotic symptoms. However, the psychotic symptom analysis had more unblinded studies and fewer studies that completed both conditions on the same day. Notwithstanding these methodological considerations, this finding suggests that acute ketamine administration is associated with more positive than negative symptoms, although the magnitude of negative symptoms associated with ketamine is still large.

### Implications for Therapeutic Use of Ketamine

Ketamine is being evaluated as a treatment for depression and some other disorders.^2,6,7^ Our findings highlight the potential risk that ketamine may induce transient positive (psychotic), negative, and other symptoms,^77^ particularly because the dose and route used to treat depression (approximately 0.5 mg/kg intravenously)^6^ is similar to those used in studies in this meta-analysis. Evidence suggests that ketamine can induce perceptual disturbances^78^ and psychotic symptoms in patients with depression (mean BPRS score, 12.6) with slightly higher positive BPRS scores than those seen in the studies included in this meta-analysis (average mean score across all BPRS studies, 7.5). People with a history of psychosis may be more vulnerable to the effects of ketamine. Our finding that using a bolus and continuous infusion method increases the effect of ketamine on psychotic symptoms highlights the importance of using slower infusions (40-60 minutes) of ketamine, an approach now adopted by some therapeutic trials.^79^

### Strengths and Limitations

Strengths of this study include the relatively large sample size and inclusion of additional data provided by authors. However, there was significant inconsistency in the summary effect sizes, suggesting variability in effects between studies. This factor can be explained in part by differences in study design, as indicated by our sensitivity findings described above. We cannot explore the effect of other methodological differences that may contribute to inconsistency, such as differences in ketamine doses or fasting status, because few studies reported sufficient detail to allow this subanalysis. The inconsistency in total symptom score may also be explained by the inclusion of different BPRS versions. A mixture of 16, 18, 20, and 24 total item scales were used, and very few studies made it clear which items they included. Consequently, individual subgroup analyses of different versions could not be conducted.

There were several important differences in exclusion criteria between the studies. In particular, most of the studies did not exclude concurrent use of psychotropic drugs,^25,29,31,32,33,34,35,36,37,38,39,40,41,52,53,54,55,56,57,58,59,60,61,80,81^ and only a few studies excluded participants with prior ketamine exposure.^30,31,42,52,53^ Although some evidence suggests that repeated ketamine exposure does not cause behavioral sensitization in humans,^82^ it would have been useful to have examined these data in more depth to determine whether these factors may alter results owing to drug tolerance or differences in subjective experience due to familiarity with prior exposure. Nevertheless, we used a random-effects model, which is a robust method of calculating the effect size when there is statistically significant inconsistency between studies.^83^

Interestingly, the blinding status did not alter the magnitude of the effect size for total, positive, or negative symptoms. Blinding participants in these experiments may be very difficult because the dissociative anesthetic effects of ketamine can be very obvious to both participant and study personnel. This possibility may further explain the heterogeneity of results because the participants’ expectations may have contributed to their drug response. Future studies could include a low dose of ketamine or active comparator, such as midazolam hydrochloride, to address this important question.

We aimed to determine ketamine’s maximal ability to induce psychotomimetic symptoms. Where symptom scales were reported at different time points, we selected the point with the highest ketamine-induced symptom score. Where this occurred, the symptom measure for the placebo group was taken at the corresponding point. Where studies included different concentrations of ketamine, we used the highest dose, again using the symptom score for the corresponding placebo condition. Therefore, the effect sizes in this study are likely to be the largest effect size seen with ketamine. Further work is thus required to better characterize the dose-response relationship and time course of ketamine’s psychotomimetic affects.

## Conclusions

We provide meta-analytic evidence that ketamine is associated with the induction of transient positive, negative, and total symptoms, with a greater increase in positive than negative symptoms in healthy volunteers. These findings support the use of ketamine as a pharmacological model of schizophrenia and, given that using a bolus plus continuous infusion method leads to greater positive psychotic symptoms, indicate that the bolus plus infusion is the best approach for this model. Ketamine is used to treat pain and for major depression. Our findings indicate a potential risk of ketamine inducing schizophreniform symptoms when it is used for these indications and that a slow infusion without bolus is preferable to minimize these risks. Further research is needed to determine the risk of these effects when ketamine is used therapeutically.

## Notes

Supplement. **eMethods 1.** Studies and Data Not Included in Meta-analysis **eMethods 2.** Newcastle-Ottawa Assessment Scale for Cohort Studies **eMethods 3.** Cochrane Tool for Assessment of Bias Within Individual Studies **eMethods 4.** Cochrane Tool for Assessment of Bias Across Studies **eMethods 5.** Refitting the Model Using *r_i_*’s Taking Values 0.1 **eMethods 6.** Refitting the Model Using *r_i_*’s Taking Values 0.7 **eMethods 7.** Comparison of the Effect of Ketamine on Positive and Negative Symptoms Using Correlation Coefficient of 0.1 **eMethods 8.** Comparison of the Effect of Ketamine on Positive and Negative Symptoms Using Correlation Coefficient of 0.7 **eMethods 9.** Heterogeneity Statistics for Subgroup Analyses **eMethods 10.** Subanalyses of Type of Symptom Scale Used **eFigure 1. **Funnel Plot for Total Symptoms **eFigure 2. **Funnel Plot for Positive Symptoms **eFigure 3. **Funnel Plot for Negative Symptoms **eFigure 4. **Subgroup Analysis of Single-Day vs Multiple-Day Studies for Total Symptoms **eFigure 5. **Subgroup Analysis of Method of Infusion (Bolus and a Continuous Infusion vs Only a Continuous Infusion) Positive Symptoms **eTable 1. **Raw Data Used in Total BPRS and PANSS Analysis for Healthy Participants **eTable 2. **Raw Data Used in Positive BPRS and PANSS Analysis for Healthy Participants **eTable 3. **Raw Data Used in Negative BPRS and PANSS Analysis for Healthy Participants **eTable 4. **Raw Data used in Total, Negative and Positive BPRS and PANSS Analysis for People With Schizophrenia **eTable 5. **Study Description, Ketamine Method, Placebo Condition, Symptoms (BPRS and PANSS), and Exclusion Criteria in Studies Examining Acute Ketamine Administration in Healthy Controls **eTable 6.** Study Description, Ketamine Administration Method, Placebo Condition, Symptoms (BPRS), and Exclusion Criteria in Studies Examining Acute Ketamine Administration to Patients With Schizophrenia **eReferences.**

## References

[1] Stevenson C Ketamine: a review. Updat Anaesth. 2005;20(20):25-29.

[2] Schwartzman RJ, Alexander GM, Grothusen JR, Paylor T, Reichenberger E, Perreault M Outpatient intravenous ketamine for the treatment of complex regional pain syndrome: a double-blind placebo controlled study. Pain. 2009;147(1-3):107-115. doi:10.1016/j.pain.2009.08.015 19783371

[3] Krystal JH, Perry EB Jr, Gueorguieva R, Comparative and interactive human psychopharmacologic effects of ketamine and amphetamine: implications for glutamatergic and dopaminergic model psychoses and cognitive function. Arch Gen Psychiatry. 2005;62(9):985-994. doi:10.1001/archpsyc.62.9.985 16143730

[4] Vollenweider FX, Geyer MA A systems model of altered consciousness: integrating natural and drug-induced psychoses. Brain Res Bull. 2001;56(5):495-507. doi:10.1016/S0361-9230(01)00646-3 11750795

[5] Krystal JH, Karper LP, Seibyl JP, Subanesthetic effects of the noncompetitive NMDA antagonist, ketamine, in humans: psychotomimetic, perceptual, cognitive, and neuroendocrine responses. Arch Gen Psychiatry. 1994;51(3):199-214. doi:10.1001/archpsyc.1994.03950030035004 8122957

[6] McGirr A, Berlim MT, Bond DJ, Fleck MP, Yatham LN, Lam RW A systematic review and meta-analysis of randomized, double-blind, placebo-controlled trials of ketamine in the rapid treatment of major depressive episodes. Psychol Med. 2015;45(4):693-704. doi:10.1017/S0033291714001603 25010396

[7] Daly EJ, Singh JB, Fedgchin M, Efficacy and safety of intranasal esketamine adjunctive to oral antidepressant therapy in treatment-resistant depression: a randomized clinical trial. JAMA Psychiatry. 2018;75(2):139-148. doi:10.1001/jamapsychiatry.2017.3739 29282469 PMC5838571

[8] Stroup DF, Berlin JA, Morton SC, Meta-analysis of observational studies in epidemiology: a proposal for reporting: Meta-analysis of Observational Studies in Epidemiology (MOOSE) group. JAMA. 2000;283(15):2008-2012. doi:10.1001/jama.283.15.2008 10789670

[9] Liberati A, Altman DG, Tetzlaff J, The PRISMA statement for reporting systematic reviews and meta-analyses of studies that evaluate health care interventions: explanation and elaboration. J Clin Epidemiol. 2009;62(10):e1-e34. doi:10.1016/j.jclinepi.2009.06.006 19631507

[10] D’Souza DC, Fridberg DJ, Skosnik PD, Dose-related modulation of event-related potentials to novel and target stimuli by intravenous Δ^9^-THC in humans. Neuropsychopharmacology. 2012;37(7):1632-1646. doi:10.1038/npp.2012.8 22334121 PMC3358754

[11] Morgan CJA, Freeman TP, Hindocha C, Schafer G, Gardner C, Curran HV Individual and combined effects of acute delta-9-tetrahydrocannabinol and cannabidiol on psychotomimetic symptoms and memory function. Transl Psychiatry. 2018;8(1):181. doi:10.1038/s41398-018-0191-x 30185793 PMC6125482

[12] Leucht S, Cipriani A, Spineli L, Comparative efficacy and tolerability of 15 antipsychotic drugs in schizophrenia: a multiple-treatments meta-analysis. Lancet. 2013;382(9896):951-962. doi:10.1016/S0140-6736(13)60733-3 23810019

[13] Nicholson IR, Chapman JE, Neufeld RWJ Variability in BPRS definitions of positive and negative symptoms. Schizophr Res. 1995;17(2):177-185. doi:10.1016/0920-9964(94)00088-P 8562492

[14] Andreasen N Scale for the Assessment of Positive Symptoms. University of Iowa; 1984.

[15] Andreasen N Scale for the Assessment of Negative Symptoms. University of Iowa; 1984.

[16] Higgins JPT, Altman DG, Gøtzsche PC, ; Cochrane Bias Methods Group; Cochrane Statistical Methods Group The Cochrane Collaboration’s tool for assessing risk of bias in randomised trials. BMJ. 2011;343(7829):d5928. doi:10.1136/bmj.d5928 22008217 PMC3196245

[17] Higgins JP, Green S, eds. *Cochrane handbook for systematic reviews of interventions* Figure 8.6.a. John Wiley & Sons; 2011. Accessed August 24, 2019. https://handbook-5-1.cochrane.org/chapter_8/figure_8_6_a_example_of_a_risk_of_bias_table_for_a_single.htm

[18] Viechtbauer W Conducting meta-analyses in R with the metafor package. J Stat Softw. 2010;36(3):1-48. doi:10.18637/jss.v036.i03

[19] McCutcheon RA, Pillinger T, Mizuno Y, The efficacy and heterogeneity of antipsychotic response in schizophrenia: a meta-analysis. Mol Psychiatry. 2019;(August):1-11. doi:10.1038/s41380-019-0502-5 31471576 PMC7610422

[20] Brugger SP, Howes OD Heterogeneity and homogeneity of regional brain structure in schizophrenia: a meta-analysis. JAMA Psychiatry. 2017;74(11):1104-1111. doi:10.1001/jamapsychiatry.2017.2663 28973084 PMC5669456

[21] Kirkham JJ, Riley RD, Williamson PR A multivariate meta-analysis approach for reducing the impact of outcome reporting bias in systematic reviews. Stat Med. 2012;31(20):2179-2195. doi:10.1002/sim.5356 22532016

[22] Bowden J, Tierney JF, Copas AJ, Burdett S Quantifying, displaying and accounting for heterogeneity in the meta-analysis of RCTs using standard and generalised Q statistics. BMC Med Res Methodol. 2011;11(1):41. doi:10.1186/1471-2288-11-41 21473747 PMC3102034

[23] Higgins JPT, Thompson SG, Deeks JJ, Altman DG Measuring inconsistency in meta-analyses. BMJ. 2003;327(7414):557-560. doi:10.1136/bmj.327.7414.557 12958120 PMC192859

[24] Egger M, Davey Smith G, Schneider M, Minder C Bias in meta-analysis detected by a simple, graphical test. BMJ. 1997;315(7109):629-634. doi:10.1136/bmj.315.7109.629 9310563 PMC2127453

[25] Malhotra AK, Pinals DA, Adler CM, Ketamine-induced exacerbation of psychotic symptoms and cognitive impairment in neuroleptic-free schizophrenics. Neuropsychopharmacology. 1997;17(3):141-150. doi:10.1016/S0893-133X(97)00036-5 9272481

[26] Lahti AC, Weiler MA, Tamara Michaelidis BA, Parwani A, Tamminga CA Effects of ketamine in normal and schizophrenic volunteers. Neuropsychopharmacology. 2001;25(4):455-467. doi:10.1016/S0893-133X(01)00243-3 11557159

[27] Malhotra AK, Breier A, Goldman D, Picken L, Pickar D The apolipoprotein E ε 4 allele is associated with blunting of ketamine-induced psychosis in schizophrenia: a preliminary report. Neuropsychopharmacology. 1998;19(5):445-448. doi:10.1016/S0893-133X(98)00031-1 9778666

[28] Kleinloog D, Uit den Boogaard A, Dahan A, Optimizing the glutamatergic challenge model for psychosis, using S+-ketamine to induce psychomimetic symptoms in healthy volunteers. J Psychopharmacol. 2015;29(4):401-413. doi:10.1177/0269881115570082 25693889

[29] Thiebes S, Leicht G, Curic S, Glutamatergic deficit and schizophrenia-like negative symptoms: new evidence from ketamine-induced mismatch negativity alterations in healthy male humans. J Psychiatry Neurosci. 2017;42(4):273-283. doi:10.1503/jpn.160187 28556775 PMC5487274

[30] Kraguljac NV, Frölich MA, Tran S, Ketamine modulates hippocampal neurochemistry and functional connectivity: a combined magnetic resonance spectroscopy and resting-state fMRI study in healthy volunteers. Mol Psychiatry. 2017;22(4):562-569. doi:10.1038/mp.2016.122 27480494 PMC5562151

[31] Newcomer JW, Farber NB, Jevtovic-Todorovic V, Ketamine-induced NMDA receptor hypofunction as a model of memory impairment and psychosis. Neuropsychopharmacology. 1999;20(2):106-118. doi:10.1016/S0893-133X(98)00067-0 9885791

[32] Krystal JH, Madonick S, Perry E, Potentiation of low dose ketamine effects by naltrexone: potential implications for the pharmacotherapy of alcoholism. Neuropsychopharmacology. 2006;31(8):1793-1800. doi:10.1038/sj.npp.1300994 16395307

[33] Grent-’t-Jong T, Rivolta D, Gross J, Acute ketamine dysregulates task-related gamma-band oscillations in thalamo-cortical circuits in schizophrenia. Brain. 2018;141(8):2511-2526. doi:10.1093/brain/awy175 30020423 PMC6061682

[34] D’Souza DC, Carson RE, Driesen N, Johannesen J, Ranganathan M, Krystal JH; Yale GlyT1 Study Group Dose-related target occupancy and effects on circuitry, behavior, and neuroplasticity of the glycine transporter-1 inhibitor pf-03463275 in healthy and schizophrenia subjects. Biol Psychiatry. 2018;84(6):413-421. doi:10.1016/j.biopsych.2017.12.019 29499855 PMC6068006

[35] Krystal JH, Karper LP, Bennett A, Interactive effects of subanesthetic ketamine and subhypnotic lorazepam in humans. Psychopharmacology (Berl). 1998;135(3):213-229. doi:10.1007/s002130050503 10463321

[36] Duncan EJ, Madonick SH, Parwani A, Clinical and sensorimotor gating effects of ketamine in normals. Neuropsychopharmacology. 2001;25(1):72-83. doi:10.1016/S0893-133X(00)00240-2 11377920

[37] Parwani A, Weiler MA, Blaxton TA, The effects of a subanesthetic dose of ketamine on verbal memory in normal volunteers. Psychopharmacology (Berl). 2005;183(3):265-274. doi:10.1007/s00213-005-0177-2 16220331

[38] Rowland LM, Astur RS, Jung RE, Bustillo JR, Lauriello J, Yeo RA Selective cognitive impairments associated with NMDA receptor blockade in humans. Neuropsychopharmacology. 2005;30(3):633-639. doi:10.1038/sj.npp.1300642 15647751

[39] Breier A, Malhotra AK, Pinals DA, Weisenfeld NI, Pickar D Association of ketamine-induced psychosis with focal activation of the prefrontal cortex in healthy volunteers. Am J Psychiatry. 1997;154(6):805-811. doi:10.1176/ajp.154.6.805 9167508

[40] van Berckel BN, Oranje B, van Ree JM, Verbaten MN, Kahn RS The effects of low dose ketamine on sensory gating, neuroendocrine secretion and behavior in healthy human subjects. Psychopharmacology (Berl). 1998;137(3):271-281. doi:10.1007/s002130050620 9683005

[41] Dickerson D, Pittman B, Ralevski E, Ethanol-like effects of thiopental and ketamine in healthy humans. J Psychopharmacol. 2010;24(2):203-211. doi:10.1177/0269881108098612 19028835 PMC4484757

[42] Abel KM, Allin MPG, Kucharska-Pietura K, Ketamine and fMRI BOLD signal: distinguishing between effects mediated by change in blood flow versus change in cognitive state. Hum Brain Mapp. 2003;18(2):135-145. doi:10.1002/hbm.10064 12518293 PMC6871893

[43] Kort NS, Ford JM, Roach BJ, Role of *N*-methyl-D-aspartate receptors in action-based predictive coding deficits in schizophrenia. Biol Psychiatry. 2017;81(6):514-524. doi:10.1016/j.biopsych.2016.06.019 27647218 PMC5203970

[44] Anand A, Charney DS, Oren DA, Attenuation of the neuropsychiatric effects of ketamine with lamotrigine: support for hyperglutamatergic effects of *N*-methyl-D-aspartate receptor antagonists. Arch Gen Psychiatry. 2000;57(3):270-276. doi:10.1001/archpsyc.57.3.270 10711913

[45] Krystal JH, D’Souza DC, Karper LP, Interactive effects of subanesthetic ketamine and haloperidol in healthy humans. Psychopharmacology (Berl). 1999;145(2):193-204. doi:10.1007/s002130051049 10463321

[46] Krystal JH, Petrakis IL, Limoncelli D, Altered NMDA glutamate receptor antagonist response in recovering ethanol-dependent patients. Neuropsychopharmacology. 2003;28(11):2020-2028. doi:10.1038/sj.npp.1300252 12888778

[47] Micallef J, Guillermain Y, Tardieu S, Effects of subanesthetic doses of ketamine on sensorimotor information processing in healthy subjects. Clin Neuropharmacol. 2002;25(2):101-106. doi:10.1097/00002826-200203000-00008 11981237

[48] Boeijinga PH, Soufflet L, Santoro F, Luthringer R Ketamine effects on CNS responses assessed with MEG/EEG in a passive auditory sensory-gating paradigm: an attempt for modelling some symptoms of psychosis in man. J Psychopharmacol. 2007;21(3):321-337. doi:10.1177/0269881107077768 17591659

[49] Höflich A, Hahn A, Küblböck M, Ketamine-induced modulation of the thalamo-cortical network in healthy volunteers as a model for schizophrenia. Int J Neuropsychopharmacol. 2015;18(9):1-11. doi:10.1093/ijnp/pyv040 25896256 PMC4576520

[50] Vernaleken I, Klomp M, Moeller O, Vulnerability to psychotogenic effects of ketamine is associated with elevated D2/3-receptor availability. Int J Neuropsychopharmacol. 2013;16(4):745-754. doi:10.1017/S1461145712000764 22906553

[51] D’Souza DC, Ahn K, Bhakta S, Nicotine fails to attenuate ketamine-induced cognitive deficits and negative and positive symptoms in humans: implications for schizophrenia. Biol Psychiatry. 2012;72(9):785-794. doi:10.1016/j.biopsych.2012.05.009 22717030

[52] Rowland LM, Beason-Held L, Tamminga CA, Holcomb HH The interactive effects of ketamine and nicotine on human cerebral blood flow. Psychopharmacology (Berl). 2010;208(4):575-584. doi:10.1007/s00213-009-1758-2 20066400 PMC2891406

[53] Stone JM, Abel KM, Allin MPG, Ketamine-induced disruption of verbal self-monitoring linked to superior temporal activation. Pharmacopsychiatry. 2011;44(1):33-48. doi:10.1055/s-0030-1267942 21154218

[54] Abdallah CG, De Feyter HM, Averill LA, The effects of ketamine on prefrontal glutamate neurotransmission in healthy and depressed subjects. Neuropsychopharmacology. 2018;43(10):2154-2160. doi:10.1038/s41386-018-0136-3 29977074 PMC6098048

[55] Passie T, Karst M, Borsutzky M, Wiese B, Emrich HM, Schneider U Effects of different subanaesthetic doses of (S)-ketamine on psychopathology and binocular depth inversion in man. J Psychopharmacol. 2003;17(1):51-56. doi:10.1177/0269881103017001698 12680739

[56] Horacek J, Brunovsky M, Novak T, Subanesthetic dose of ketamine decreases prefrontal theta cordance in healthy volunteers: implications for antidepressant effect. Psychol Med. 2010;40(9):1443-1451. doi:10.1017/S0033291709991619 19995475

[57] Morgan HL, Turner DC, Corlett PR, Exploring the impact of ketamine on the experience of illusory body ownership. Biol Psychiatry. 2011;69(1):35-41. doi:10.1016/j.biopsych.2010.07.032 20947068 PMC3025328

[58] Powers AR III, Gancsos MG, Finn ES, Morgan PT, Corlett PR Ketamine-induced hallucinations. Psychopathology. 2015;48(6):376-385. doi:10.1159/000438675 26361209 PMC4684980

[59] Nagels A, Kirner-Veselinovic A, Krach S, Kircher T Neural correlates of S-ketamine induced psychosis during overt continuous verbal fluency. Neuroimage. 2011;54(2):1307-1314. doi:10.1016/j.neuroimage.2010.08.021 20727411

[60] Driesen NR, McCarthy G, Bhagwagar Z, Relationship of resting brain hyperconnectivity and schizophrenia-like symptoms produced by the NMDA receptor antagonist ketamine in humans. Mol Psychiatry. 2013;18(11):1199-1204. doi:10.1038/mp.2012.194 23337947 PMC3646075

[61] Mathalon DH, Ahn K-H, Perry EBJ Jr, Effects of nicotine on the neurophysiological and behavioral effects of ketamine in humans. Front Psychiatry. 2014;5:3. doi:10.3389/fpsyt.2014.00003 24478731 PMC3900858

[62] Morrison RL, Fedgchin M, Singh J, Effect of intranasal esketamine on cognitive functioning in healthy participants: a randomized, double-blind, placebo-controlled study. Psychopharmacology (Berl). 2018;235(4):1107-1119. doi:10.1007/s00213-018-4828-5 29392371 PMC5869899

[63] van de Loo AJAE, Bervoets AC, Mooren L, The effects of intranasal esketamine (84 mg) and oral mirtazapine (30 mg) on on-road driving performance: a double-blind, placebo-controlled study. Psychopharmacology (Berl). 2017;234(21):3175-3183. doi:10.1007/s00213-017-4706-6 28755104 PMC5660834

[64] Lahti AC, Holcomb HH, Medoff DR, Tamminga CA Ketamine activates psychosis and alters limbic blood flow in schizophrenia. Neuroreport. 1995;6(6):869-872. doi:10.1097/00001756-199504190-00011 7612873

[65] Lahti AC, Koffel B, LaPorte D, Tamminga CA Subanesthetic doses of ketamine stimulate psychosis in schizophrenia. Neuropsychopharmacology. 1995;13(1):9-19. doi:10.1016/0893-133X(94)00131-I 8526975

[66] Holcomb HH, Lahti AC, Medoff DR, Cullen T, Tamminga CA Effects of noncompetitive NMDA receptor blockade on anterior cingulate cerebral blood flow in volunteers with schizophrenia. Neuropsychopharmacology. 2005;30(12):2275-2282. doi:10.1038/sj.npp.1300824 16034443

[67] Medoff DR, Holcomb HH, Lahti AC, Tamminga CA Probing the human hippocampus using rCBF: contrasts in schizophrenia. Hippocampus. 2001;11(5):543-550. doi:10.1002/hipo.1070 11732707

[68] LaPorte DJ, Lahti AC, Koffel B, Tamminga CA Absence of ketamine effects on memory and other cognitive functions in schizophrenia patients. J Psychiatr Res. 1996;30(5):321-330. doi:10.1016/0022-3956(96)00018-0 8923336

[69] Lahti AC, Warfel D, Michaelidis T, Weiler MA, Frey K, Tamminga CA Long-term outcome of patients who receive ketamine during research. Biol Psychiatry. 2001;49(10):869-875. doi:10.1016/S0006-3223(00)01037-4 11343683

[70] Malhotra AK, Adler CM, Kennison SD, Elman I, Pickar D, Breier A Clozapine blunts *N*-methyl-D-aspartate antagonist-induced psychosis: a study with ketamine. Biol Psychiatry. 1997;42(8):664-668. doi:10.1016/S0006-3223(96)00546-X 9325559

[71] Leucht S, Kane JM, Kissling W, Hamann J, Etschel E, Engel RR What does the PANSS mean? Schizophr Res. 2005;79(2-3):231-238. doi:10.1016/j.schres.2005.04.008 15982856

[72] Farber NB, Wozniak DF, Price MT, Age-specific neurotoxicity in the rat associated with NMDA receptor blockade: potential relevance to schizophrenia? Biol Psychiatry. 1995;38(12):788-796. doi:10.1016/0006-3223(95)00046-1 8750036

[73] Morgan CJA, Perry EB, Cho H-S, Krystal JH, D’Souza DC Greater vulnerability to the amnestic effects of ketamine in males. Psychopharmacology (Berl). 2006;187(4):405-414. doi:10.1007/s00213-006-0409-0 16896964

[74] Jevtovic-Todorovic V, Wozniak DF, Benshoff ND, Olney JW A comparative evaluation of the neurotoxic properties of ketamine and nitrous oxide. Brain Res. 2001;895(1-2):264-267. doi:10.1016/S0006-8993(01)02079-0 11259788

[75] Winters WD, Hance AJ, Cadd GC, Lakin ML Seasonal and sex influences on ketamine-induced analgesia and catalepsy in the rat: a possible role for melatonin. Neuropharmacology. 1986;25(10):1095-1101. doi:10.1016/0028-3908(86)90156-5 3785578

[76] Schulz KF, Altman DG, Moher D; CONSORT Group CONSORT 2010 statement: updated guidelines for reporting parallel group randomised trials. BMC Med. 2010;8(1):18. doi:10.1186/1741-7015-8-18 20334633 PMC2860339

[77] Perry EB Jr, Cramer JA, Cho H-S, ; Yale Ketamine Study Group Psychiatric safety of ketamine in psychopharmacology research. Psychopharmacology (Berl). 2007;192(2):253-260. doi:10.1007/s00213-007-0706-2 17458544

[78] Zarate CA Jr, Singh JB, Carlson PJ, A randomized trial of an *N*-methyl-D-aspartate antagonist in treatment-resistant major depression. Arch Gen Psychiatry. 2006;63(8):856-864. doi:10.1001/archpsyc.63.8.856 16894061

[79] Fond G, Loundou A, Rabu C, Ketamine administration in depressive disorders: a systematic review and meta-analysis. Psychopharmacology (Berl). 2014;231(18):3663-3676. doi:10.1007/s00213-014-3664-5 25038867

[80] Musso F, Brinkmeyer J, Ecker D, Ketamine effects on brain function—simultaneous fMRI/EEG during a visual oddball task. Neuroimage. 2011;58(2):508-525. doi:10.1016/j.neuroimage.2011.06.045 21723949

[81] Umbricht D, Schmid L, Koller R, Vollenweider FX, Hell D, Javitt DC Ketamine-induced deficits in auditory and visual context-dependent processing in healthy volunteers: implications for models of cognitive deficits in schizophrenia. Arch Gen Psychiatry. 2000;57(12):1139-1147. doi:10.1001/archpsyc.57.12.1139 11115327

[82] Cho H-S, D’Souza DC, Gueorguieva R, Absence of behavioral sensitization in healthy human subjects following repeated exposure to ketamine. Psychopharmacology (Berl). 2005;179(1):136-143. doi:10.1007/s00213-004-2066-5 15682309

[83] Cheung MW-L, Cheung SF Random-effects models for meta-analytic structural equation modeling: review, issues, and illustrations. Res Synth Methods. 2016;7(2):140-155. doi:10.1002/jrsm.1166 27286900

