# Supplementary material for: Association of Ketamine With Psychiatric Symptoms and Implications for Its Therapeutic Use and for Understanding Schizophrenia: A Systematic Review and Meta-analysis
Source: JAMA Netw Open. 2020 May 21;3(5):e204693. doi: 10.1001/jamanetworkopen.2020.4693 (PMC7243091; doi:10.1001/jamanetworkopen.2020.4693)
Supplement: Supplement. — eMethods 1. Studies and Data Not Included in Meta-analysis eMethods 2. Newcastle-Ottawa Assessment Scale for Cohort Studies eMethods 3. Cochrane Tool for Assessment of Bias Within Individual Studies eMethods 4. Cochrane Tool for Assessment of Bias Across Studies eMethods 5. Refitting the Model Using ri’s Taking Values 0.1 eMethods 6. Refitting the Model Using ri’s Taking Values 0.7 eMethods 7. Comparison of the Effect of Ketamine on Positive and Negative Symptoms Using Correlation Coefficient of 0.1 eMethods 8. Comparison of the Effect of Ketamine on Positive and Negative Symptoms Using Correlation Coefficient of 0.7 eMethods 9. Heterogeneity Statistics for Subgroup Analyses eMethods 10. Subanalyses of Type of Symptom Scale Used eFigure 1. Funnel Plot for Total Symptoms eFigure 2. Funnel Plot for Positive Symptoms eFigure 3. Funnel Plot for Negative Symptoms eFigure 4. Subgroup Analysis of Single-Day vs Multiple-Day Studies for Total Symptoms eFigure 5. Subgroup Analysis of Method of Infusion (Bolus and a Continuous Infusion vs Only a Continuous Infusion) Positive Symptoms eTable 1. Raw Data Used in Total BPRS and PANSS Analysis for Healthy Participants eTable 2. Raw Data Used in Positive BPRS and PANSS Analysis for Healthy Participants eTable 3. Raw Data Used in Negative BPRS and PANSS Analysis for Healthy Participants eTable 4. Raw Data used in Total, Negative and Positive BPRS and PANSS Analysis for People With Schizophrenia eTable 5. Study Description, Ketamine Method, Placebo Condition, Symptoms (BPRS and PANSS), and Exclusion Criteria in Studies Examining Acute Ketamine Administration in Healthy Controls eTable 6. Study Description, Ketamine Administration Method, Placebo Condition, Symptoms (BPRS), and Exclusion Criteria in Studies Examining Acute Ketamine Administration to Patients With Schizophrenia eReferences. [file jamanetwopen-3-e204693-s001.pdf]

Supplementary Online Content

Beck K, Hindley G, Borgan F, et al. Association of ketamine with psychiatric symptoms and implications for its therapeutic use and for understanding schizophrenia: a systematic review and meta-analysis. *JAMA Netw Open*. 2020;3(5):e204693. doi:10.1001/jamanetworkopen.2020.4693

eMethods 1. Studies and Data Not Included in Meta-analysis
eMethods 2. Newcastle-Ottawa Assessment Scale for Cohort Studies
eMethods 3. Cochrane Tool for Assessment of Bias Within Individual Studies
eMethods 4. Cochrane Tool for Assessment of Bias Across Studies
eMethods 5. Refitting the Model Using r_i's Taking Values 0.1
eMethods 6. Refitting the Model Using r_i's Taking Values 0.7
eMethods 7. Comparison of the Effect of Ketamine on Positive and Negative Symptoms Using Correlation Coefficient of 0.1
eMethods 8. Comparison of the Effect of Ketamine on Positive and Negative Symptoms Using Correlation Coefficient of 0.7
eMethods 9. Heterogeneity Statistics for Subgroup Analyses
eMethods 10. Subanalyses of Type of Symptom Scale Used
eFigure 1. Funnel Plot for Total Symptoms
eFigure 2. Funnel Plot for Positive Symptoms
eFigure 3. Funnel Plot for Negative Symptoms
eFigure 4. Subgroup Analysis of Single-Day vs Multiple-Day Studies for Total Symptoms
eFigure 5. Subgroup Analysis of Method of Infusion (Bolus and a Continuous Infusion vs Only a Continuous Infusion) Positive Symptoms
eTable 1. Raw Data Used in Total BPRS and PANSS Analysis for Healthy Participants
eTable 2. Raw Data Used in Positive BPRS and PANSS Analysis for Healthy Participants
eTable 3. Raw Data Used in Negative BPRS and PANSS Analysis for Healthy Participants
eTable 4. Raw Data used in Total, Negative and Positive BPRS and PANSS Analysis for People With Schizophrenia
eTable 5. Study Description, Ketamine Method, Placebo Condition, Symptoms (BPRS and PANSS), and Exclusion Criteria in Studies Examining Acute Ketamine Administration in Healthy Controls
eTable 6. Study Description, Ketamine Administration Method, Placebo Condition, Symptoms (BPRS), and Exclusion Criteria in Studies Examining Acute Ketamine Administration to Patients With Schizophrenia
eReferences.

This supplementary material has been provided by the authors to give readers additional information about their work.

©2020 Beck K et al. *JAMA Network Open*.

eMethods 1. Studies and Data Not Included in Meta-analysis

Effect of ketamine administration to healthy controls: meta-analysis
28 studies[1,2,11–17,3–10] [18] met inclusion but were excluded as they were identified as using data that overlapped with studies that were already included in the meta-analysis. 1 study[19] met inclusion criteria but was excluded from the positive symptom meta-analysis as they were identified as using data that overlapped with other studies already included.

18 studies [15,20,29–36,21–28] did not respond to two requests for data prior to submission.
[37–45] replied to the request for data but were unable to provide raw data from their studies, as it was not available.

3 studies [46–48] replied to the first request for data but were not able to provide data by the time of submission.

Exclusion based on symptom items (meta-analysis of positive and negative symptoms):

Studies that appeared to have data for the positive and negative subscales but had not specified the symptom items included – such that we were not able to determine if the study met inclusion criteria for this part of the meta-analysis were emailed to request the items they included. If studies had data for the total symptoms these studies were also emailed to determine if they had raw data for the positive and symptom subscales

8 studies [49–55] were included in the meta-analysis but were not specifically included in the positive symptom analysis as they did not clearly include the items included in the inclusion criteria.

3 Studies [51–53,55] were included in the meta-analysis but were not specifically included in the negative symptom analysis as they did not clearly include the items included in the inclusion criteria.

1 study [56] was not included in the meta-analysis, this study only included information on positive symptoms and there was no information on the items included.

1 study [57] had a score of 0 for both the S.Ds for the ketamine and control condition for positive symptoms, therefore, this was excluded as the effect size could not be calculated in this situation.

The effects of ketamine in people with schizophrenia
1 study [58] met inclusion criteria but was excluded after author confirmed by email that there was sample overlap with other included studies.

1 study [59] had positive and negative symptom data removed from analysis because author confirmed by email that there was sample overlap with other included studies. Where there was overlap the study with the largest sample size was included.

©2020 Beck K et al. *JAMA Network Open*.

6 studies [18,31,60–63] all from one group met inclusion criteria but were excluded due to the possibility that there was a sample overlap with a study already included. Information was requested from the author by email.

©2020 Beck K et al. *JAMA Network Open*.

eMethods 2. Newcastle-Ottawa Assessment Scale for Cohort Studies

		**1**	**2**	**3**	**4**	**5**	**6**	**7**	**8**
Kraguljac	2017	unclear	*	*	*	**		*	*
Kort	2017	unclear	*	*	*	*	*	*	*
Duncan	2001	All male	*	*	*	*	*	*	*
Parwani	2005	unclear	*	*	*	*	*	*	*
Rowland	2005	All male	*	*	*	*	*	*	*
Abel	2003	All male	*	*		**	*	*	*
Anand	2000	*	*	*	*	*	*	*	*
Krystal	1998	*	*	*	*	*	*	*	*
Breier	1997	*	*	*		*	*	*	*
Van Berckel	1998	All male	*	*	*	*	*	*	*
Malhotra	1997	*	*	*	*	*	*	*	*
Krystal	1999	*	*	*	*	*	*	*	*
Krystal	2003	*	*	*	*	*	*	*	*
Micallef	2002	22-33 years	*	*	*	*	*	*	*
Rowland	2010	unclear	*	*	*	*	*	*	*
Newcomer	1999	All male	*	*	*	**	*	*	
Stone	2011	All male	*	*		**	*	*	*
Boeijinga	2007	All male	*	*	*	*	*	*	*
Passie	2003	All male	*	*		*	*	*	*
Abdallah	2018	unclear	*	*		*		*	*
Morgan	2011	unclear	*	*		*	*	*	*
Horacek	2010	*	*	*	*	*	*	*	*
Thiebes	2017	All male	*	*	*	*		*	
Powers	2015	unclear	*	*		*		*	*
Mathalon	2014	*	*	*	*	*	*	*	unclear
Hoflich	2015	unclear	*	*	*	*	*	*	*
Nagels	2011	All male	*	*	*	*	*	*	*
Driesen	2013	*	*	*	*	*		*	*
Vernaleken	2013	All male	*	*	*	*		*	*
Krystal	2005	unclear	*	*	*	*	*	*	*
Krystal	2006	unclear	*	*	*	*	*	*	*
Kleinloog	2015	*	*	*		*	*	*	*
D'Souza	2012	unclear	*	*		*	*	*	*
Grent-'t-Jong	2018	*	*	*	*	*		*	*
D'Souza	2018	*	*	*	*	*	*	*	*
Dickerson	2010	unclear	*	*		*		*	unclear

©2020 Beck K et al. *JAMA Network Open*.

1) **Representativeness of the exposed cohort:** * = "truly representative of average healthy individual in the community" or "somewhat representative of the average healthy individual in the community"
2) **Selection of the non-exposed cohort**:* = "Drawn from the same community as the exposed cohort" (within person design)
3) **Ascertainment of exposure:** * = "Secure record" or "Structured interview" (exposure provided as part of an experiment)
4) **Demonstration that outcome of interest was not present at start of study:** * = "yes" i.e SCID used to screen out people with significant psychopathology
5) **Comparability of cohorts on the basis of the design or analysis:**
    a. * = study controls for prior ketamine use
    b. * = study controls for additional factor/factors
6) **Assessment of outcome:** * = "independent blind assessment" or "record linkage"
7) **Was a follow-up long enough for outcomes to occur**: * = "yes" ie there was sufficient timepoints to show peak psychopathology
8) **Adequacy of follow-up cohorts:** * = "Complete follow-up – all subjects accounted for" or subjects lost to follow-up unlikely to introduce bias – number lost <20% or description provide of those lost"

©2020 Beck K et al. *JAMA Network Open*.

eMethods 3. Cochrane Tool for Assessment of Bias Within Individual Studies

Study	Selection bias		Performance bias	Detection bias	Attrition bias	Reporting bias
	Random sequence generation	Allocation concealment	Blinding of participants and personnel	Blinding of outcome assessment	Incomplete outcome data	Selective reporting
Kraguljac 2017	High risk- unblinded	High risk- unblinded	High risk - unblinded	High risk- unblinded	Low risk- no missing data	Low risk – outcomes clear
Kort 2017	Unclear	Unclear	Low risk	Low risk	Low risk – no missing data	Low risk – outcomes clear
Duncan 2001	Unclear	Unclear	Low risk	Low risk	Low risk – no missing data	Low risk- outcomes clear
Parwani 2005	Unclear	Unclear	Low risk	Low risk	Low risk – no missing data	Low risk- outcomes clear
Rowland 2005	Unclear	Unclear	Low risk	Low risk	Low risk – no missing data	Low risk- outcomes clear
Abel 2003	Unclear	Unclear	Low risk	Low risk	Low risk – no missing data	Low risk- outcomes clear
Anand 2000	Unclear	Unclear	Low risk	Low risk	Low risk - <20% drop outs and missing outcome data balanced in numbers across intervention groups, with similar reasons for missing data across groups	Low risk- outcomes clear
Krystal 1998	Unclear	Unclear	Low risk	Low risk	Low risk – no missing data	Low risk- outcomes clear
Breier 1997	Unclear	Unclear	Low risk	Low risk	Low risk - <20% drop outs and missing outcome data balanced in numbers across intervention groups, with similar reasons for missing data across groups	Low risk- outcomes clear
Van Berckel 1998	Unclear	Unclear	Low risk	Low risk	Low risk - <20% drop outs and missing outcome data balanced in numbers across intervention groups, with similar reasons for missing data across groups	Low risk- outcomes clear
Malhotra 1997	Unclear	Unclear	Low risk	Low risk	Low risk – no missing data	Low risk- outcomes clear
Krystal 1999	Unclear	Unclear	Low risk	Low risk	Low risk - <20% drop outs and missing outcome data balanced in numbers across intervention groups, with similar reasons for	Low risk- outcomes clear

©2020 Beck K et al. *JAMA Network Open*.

					missing data across groups	
Krystal 2003	Unclear	Unclear	Low risk	Low risk	Low risk - <20% drop outs and missing outcome data balanced in numbers across intervention groups, with similar reasons for missing data across groups	Low risk- outcomes clear
Micallef 2002	Unclear	Unclear	Low risk	Low risk	Low risk – no missing data	Low risk- outcomes clear
Rowland 2010	Unclear	Unclear	Low risk	Low risk	Low risk – no missing data	Low risk- outcomes clear
Newcomer 1999	Unclear	Unclear	Low risk	Low risk	Low risk – no missing data	Low risk- outcomes clear
Stone 2011	Unclear	Unclear	Low risk	Low risk	Low risk - <20% drop outs and missing outcome data balanced in numbers across intervention groups, with similar reasons for missing data across groups	Low risk- outcomes clear
Boeijinga 2007	Unclear	Unclear	Low risk	Low risk	Low risk – no missing data	Low risk- outcomes clear
Passie 2003	Unclear	Unclear	Low risk	Low risk	Low risk – no missing data	Low risk- outcomes clear
Abdallah 2018	High risk- single blind	High risk-single blind	High risk -single blind	High risk -single blind	Low risk - <20% drop outs and missing outcome data balanced in numbers across intervention groups, with similar reasons for missing data across groups	Low risk- outcomes clear
Morgan 2011	High risk – not randomized	High risk – not randomized	Low risk	Low risk	Low risk – no missing data	Low risk- outcomes clear
Horacek 2010	Unclear	Unclear	Low risk	Low risk	Low risk – no missing data	Low risk- outcomes clear
Thiebes 2017	High risk- single blind	High risk-single blind	High risk-single blind	High risk-single blind	Low risk – no missing data	Low risk- outcomes clear
Powers 2015	High risk- unblinded	High risk- unblinded	High risk- unblinded	High risk- unblinded	Low risk - <20% drop outs and missing outcome data balanced in numbers across intervention groups, with similar reasons for missing data across groups	Low risk- outcomes clear
Mathalon 2014	Unclear	Unclear	Low risk	Low risk	Low risk – unclear how many drop outs occurred but outcome data balanced in numbers across intervention groups, with similar reasons for missing data across groups	Low risk- outcomes clear

©2020 Beck K et al. *JAMA Network Open*.

Hoflich 2015	Unclear	Unclear	Low risk	Low risk	Low risk – unclear how many drop outs occurred but outcome data balanced in numbers across intervention groups, with similar reasons for missing data across groups	Low risk- outcomes clear
Nagels 2011	Unclear	Unclear	Low risk	Low risk	Low risk – no missing data	Low risk- outcomes clear
Driesen 2013	High risk- unblinded	High risk- unblinded	High risk- unblinded	High risk- unblinded	Low risk – no missing data	Low risk- outcomes clear
Vernaleken 2013	High risk- single blind	High risk-single blind	High risk-single blind	High risk-single blind	Low risk – no missing data	Low risk- outcomes clear
Krystal 2005	Unclear	Unclear	Low risk	Low risk	Low risk – unclear how many drop outs occurred but outcome data balanced in numbers across intervention groups, with similar reasons for missing data across groups	Low risk- outcomes clear
Krystal 2006	Unclear	Unclear	Low risk	Low risk	Low risk –<20% drop outs, missing outcome data balanced in numbers across intervention groups, with similar reasons for missing data across groups	Low risk- outcomes clear
Kleinloog 2015	Unclear	Unclear	Low risk	Low risk	Low risk – no missing data	Low risk- outcomes clear
D'Souza 2012	Unclear	Unclear	Low risk	Low risk	Low risk - <20% drop outs and missing outcome data balanced in numbers across intervention groups, with similar reasons for missing data across groups	Low risk- outcomes clear
Grent-'t-Jong 2018	High risk- single blind	High risk-single blind	High risk-single blind	High risk-single blind	Low risk – no missing data	Low risk- outcomes clear
D'Souza 2018	Unclear - blinding status not recorded	Unclear - blinding status not recorded	Unclear - blinding status not recorded	Unclear -blinding status not recorded	Low risk - <20% drop outs and missing outcome data balanced in numbers across intervention groups, with similar reasons for missing data across groups	Low risk- outcomes clear
Dickerson 2010	High risk- single blind	High risk-single blind	High risk-single blind	High risk-single blind	Unclear	Low risk- outcomes clear

Cochrane risk of bias scoring template [64]

©2020 Beck K et al. *JAMA Network Open*.

**RANDOM SEQUENCE GENERATION**<br><br>**Selection bias (biased allocation to interventions) due to inadequate generation of a randomised sequence.**	
Criteria for a judgement of 'Low risk' of bias.	The investigators describe a random component in the sequence generation process such as:<br><br>• Referring to a random number table;<br>• Using a computer random number generator;<br>• Coin tossing;<br>• Shuffling cards or envelopes;<br>• Throwing dice;<br>• Drawing of lots;<br>• Minimization*.<br><br>*Minimization may be implemented without a random element, and this is considered to be equivalent to being random.
Criteria for the judgement of 'High risk' of bias.	The investigators describe a non-random component in the sequence generation process. Usually, the description would involve some systematic, non-random approach, for example:<br><br>• Sequence generated by odd or even date of birth;<br>• Sequence generated by some rule based on date (or day) of admission;<br>• Sequence generated by some rule based on hospital or clinic record number.<br><br>Other non-random approaches happen much less frequently than the systematic approaches mentioned above and tend to be obvious. They usually involve judgement or some method of non-random categorization of participants, for example:<br><br>• Allocation by judgement of the clinician;<br>• Allocation by preference of the participant;<br>• Allocation based on the results of a laboratory test or a series of tests;<br>• Allocation by availability of the intervention.
Criteria for the judgement of 'Unclear risk' of bias.	Insufficient information about the sequence generation process to permit judgement of 'Low risk' or 'High risk'.
**ALLOCATION CONCEALMENT**<br><br>**Selection bias (biased allocation to interventions) due to inadequate concealment of allocations prior to assignment.**	
Criteria for a judgement of 'Low risk' of bias.	Participants and investigators enrolling participants could not foresee assignment because one of the following, or an equivalent method, was used to conceal allocation:<br><br>• Central allocation (including telephone, web-based and pharmacy-controlled randomization);

©2020 Beck K et al. *JAMA Network Open*.

	• Sequentially numbered drug containers of identical appearance;<br>• Sequentially numbered, opaque, sealed envelopes.
Criteria for the judgement of 'High risk' of bias.	Participants or investigators enrolling participants could possibly foresee assignments and thus introduce selection bias, such as allocation based on:<br><br>• Using an open random allocation schedule (e.g. a list of random numbers);<br>• Assignment envelopes were used without appropriate safeguards (e.g. if envelopes were unsealed or nonopaque or not sequentially numbered);<br>• Alternation or rotation;<br>• Date of birth;<br>• Case record number;<br>• Any other explicitly unconcealed procedure.
Criteria for the judgement of 'Unclear risk' of bias.	Insufficient information to permit judgement of 'Low risk' or 'High risk'. This is usually the case if the method of concealment is not described or not described in sufficient detail to allow a definite judgement – for example if the use of assignment envelopes is described, but it remains unclear whether envelopes were sequentially numbered, opaque and sealed.
**BLINDING OF PARTICIPANTS AND PERSONNEL**<br><br>**Performance bias due to knowledge of the allocated interventions by participants and personnel during the study.**	
Criteria for a judgement of 'Low risk' of bias.	Any one of the following:<br><br>• No blinding or incomplete blinding, but the review authors judge that the outcome is not likely to be influenced by lack of blinding;<br>• Blinding of participants and key study personnel ensured, and unlikely that the blinding could have been broken.
Criteria for the judgement of 'High risk' of bias.	Any one of the following:<br><br>• No blinding or incomplete blinding, and the outcome is likely to be influenced by lack of blinding;<br>• Blinding of key study participants and personnel attempted, but likely that the blinding could have been broken, and the outcome is likely to be influenced by lack of blinding.
Criteria for the judgement of 'Unclear risk' of bias.	Any one of the following:<br><br>• Insufficient information to permit judgement of 'Low risk' or 'High risk';<br>• The study did not address this outcome.
**BLINDING OF OUTCOME ASSESSMENT**<br><br>**Detection bias due to knowledge of the allocated interventions by outcome assessors.**	
Criteria for a judgement of 'Low risk' of bias.	Any one of the following:

©2020 Beck K et al. *JAMA Network Open*.

	• No blinding of outcome assessment, but the review authors judge that the outcome measurement is not likely to be influenced by lack of blinding;<br>• Blinding of outcome assessment ensured, and unlikely that the blinding could have been broken.
Criteria for the judgement of 'High risk' of bias.	Any one of the following:<br><br>• No blinding of outcome assessment, and the outcome measurement is likely to be influenced by lack of blinding;<br>• Blinding of outcome assessment, but likely that the blinding could have been broken, and the outcome measurement is likely to be influenced by lack of blinding.
Criteria for the judgement of 'Unclear risk' of bias.	Any one of the following:<br><br>• Insufficient information to permit judgement of 'Low risk' or 'High risk';<br>• The study did not address this outcome.
**INCOMPLETE OUTCOME DATA**<br><br>**Attrition bias due to amount, nature or handling of incomplete outcome data.**	
Criteria for a judgement of 'Low risk' of bias.	Any one of the following:<br><br>• No missing outcome data;<br>• Reasons for missing outcome data unlikely to be related to true outcome (for survival data, censoring unlikely to be introducing bias);<br>• Missing outcome data balanced in numbers across intervention groups, with similar reasons for missing data across groups;<br>• For dichotomous outcome data, the proportion of missing outcomes compared with observed event risk not enough to have a clinically relevant impact on the intervention effect estimate;<br>• For continuous outcome data, plausible effect size (difference in means or standardized difference in means) among missing outcomes not enough to have a clinically relevant impact on observed effect size;<br>• Missing data have been imputed using appropriate methods.
Criteria for the judgement of 'High risk' of bias.	Any one of the following:<br><br>• Reason for missing outcome data likely to be related to true outcome, with either imbalance in numbers or reasons for missing data across intervention groups;<br>• For dichotomous outcome data, the proportion of missing outcomes compared with observed event risk enough to induce clinically relevant bias in intervention effect estimate;<br>• For continuous outcome data, plausible effect size (difference in means or standardized difference in means) among missing outcomes enough to induce clinically relevant bias in observed effect size;<br>• 'As-treated' analysis done with substantial departure of the intervention received from that assigned at randomization;

©2020 Beck K et al. *JAMA Network Open*.

	• Potentially inappropriate application of simple imputation.
Criteria for the judgement of 'Unclear risk' of bias.	Any one of the following:<br><br>• Insufficient reporting of attrition/exclusions to permit judgement of 'Low risk' or 'High risk' (e.g. number randomized not stated, no reasons for missing data provided);<br>• The study did not address this outcome.
**SELECTIVE REPORTING**<br><br>**Reporting bias due to selective outcome reporting.**	
Criteria for a judgement of 'Low risk' of bias.	Any of the following:<br><br>• The study protocol is available and all of the study's pre-specified (primary and secondary) outcomes that are of interest in the review have been reported in the pre-specified way;<br>• The study protocol is not available but it is clear that the published reports include all expected outcomes, including those that were pre-specified (convincing text of this nature may be uncommon).
Criteria for the judgement of 'High risk' of bias.	Any one of the following:<br><br>• Not all of the study's pre-specified primary outcomes have been reported;<br>• One or more primary outcomes is reported using measurements, analysis methods or subsets of the data (e.g. subscales) that were not pre-specified;<br>• One or more reported primary outcomes were not pre-specified (unless clear justification for their reporting is provided, such as an unexpected adverse effect);<br>• One or more outcomes of interest in the review are reported incompletely so that they cannot be entered in a meta-analysis;<br>• The study report fails to include results for a key outcome that would be expected to have been reported for such a study.
Criteria for the judgement of 'Unclear risk' of bias.	Insufficient information to permit judgement of 'Low risk' or 'High risk'. It is likely that the majority of studies will fall into this category.
**OTHER BIAS**<br><br>**Bias due to problems not covered elsewhere in the table.**	
Criteria for a judgement of 'Low risk' of bias.	The study appears to be free of other sources of bias.
Criteria for the judgement of 'High risk' of bias.	There is at least one important risk of bias. For example, the study:<br><br>• Had a potential source of bias related to the specific study design used; or<br>• Has been claimed to have been fraudulent; or

©2020 Beck K et al. *JAMA Network Open*.

	• Had some other problem.
Criteria for the judgement of 'Unclear risk' of bias.	There may be a risk of bias, but there is either:<br><br>• Insufficient information to assess whether an important risk of bias exists; or<br>• Insufficient rationale or evidence that an identified problem will introduce bias.

©2020 Beck K et al. *JAMA Network Open*.

eMethods 4. Cochrane Tool for Assessment of Bias Across Studies

	Type of bias	Risk of bias across all studies
Random sequence generation	Selection bias	**Unclear risk of bias.** Mostly this is unclear across the studies. However, those that have the required information documented to make a decision are high risk.<br><br>Therefore there is plausible bias that raises some doubt about the results.
Allocation concealment	Selection bias	**Unclear risk of bias.** Mostly this is unclear across the studies. However, those that have the required information documented to make a decision are high risk.<br><br>Therefore there is plausible bias that raises some doubt about the results.
Blinding of participants and personnel	Performance bias	Low risk of bias. Plausible bias unlikely to seriously alter the results.
Blinding of outcome assessment	Detection bias	Low risk of bias. Plausible bias unlikely to seriously alter the results.
Incomplete outcome data	Attrition bias	Low risk of bias. Plausible bias unlikely to seriously alter the results.
Selective reporting	Reporting bias	Low risk of bias. Plausible bias unlikely to seriously alter the results.

©2020 Beck K et al. *JAMA Network Open*.

eMethods 5. Refitting the Model Using r_i's Taking Values 0.1

Acute administration of ketamine generated an increase in psychopathology in healthy participants, seen as a statistically significant increase in the total symptoms (SMD = 1.37, 95% CI, 1.12 to 1.63, P<0.001), positive symptoms (SMD = 1.42, 95% CI, 1.19 to 1.66, P<0.001) and negative symptoms (SMD =1.04, 95% CI, 0.85 to 1.23, P<0.001) compared with a placebo condition.

eMethods 6. Refitting the Model Using r_i's Taking Values 0.7

Acute administration of ketamine generated an increase in psychopathology in healthy participants, seen as a statistically significant increase in the total symptoms (SMD = 1.59, 95% CI, 1.30 to 1.88 , P= <0.001), positive symptoms (SMD = 1.64, 95% CI, 1.35 to 1.93, P< 0.001) and negative symptoms (Standardised mean change score =1.24, 95% CI, 1.04 to 1.44, P<0.001) compared with a placebo condition.

eMethods 7. Comparison of the Effect of Ketamine on Positive and Negative Symptoms Using Correlation Coefficient of 0.1

A comparison of effect sizes demonstrated that ketamine has a greater effect on positive symptoms when compared with negative symptoms (Estimate 0.37, 95% CI=0.13 to 0.61, p=0.003).

eMethods 8. Comparison of the Effect of Ketamine on Positive and Negative Symptoms Using Correlation Coefficient of 0.7

A comparison of effect sizes demonstrated that ketamine has a greater effect on positive symptoms when compared with negative symptoms (Estimate 0.36, 95% CI=0.11 to 0.61, p=0.004).

©2020 Beck K et al. *JAMA Network Open*.

eMethods 9. Heterogeneity Statistics for Subgroup Analyses

Total psychopathology

Ketamine preparation
I^2 value for racemic ketamine preparation is 73.86%
I^2 value for s-ketamine preparation is 80.34%

Blinding method
I^2 value for double blind studies is 70.60%
I^2 value for unblinded/single blind studies is 83.50%

Infusion method
I^2 value for the studies including the bolus followed by continuous infusion method is 79.43%
I^2 value for the studies including the continuous infusion alone method is 50.73%

Single-day versus multiple-day studies
I^2 value for single-day studies is 0%
I^2 value for multiple-day studies is 63.38%.

Positive psychotic symptoms

Ketamine preparation
I^2 value for racemic ketamine preparation is 78.28%
I^2 value for s-ketamine preparation is 63.81%

Blinding method
I^2 value for double blind studies is 79.48%
I^2 value for unblinded/single blind studies is 60.96%

Infusion method
I^2 value for the studies including the bolus followed by continuous infusion method is 72.38%.
I^2 value for the studies including the continuous infusion alone method is 27.71%

Single-day versus multiple-day studies
I^2 value for single-day studies is 0%
I^2 value for multiple-day studies is 83.00%

Negative psychotic symptoms

Ketamine preparation
I^2 value for racemic ketamine preparation is 67.24%
I^2 value for s-ketamine preparation is 59.06%

©2020 Beck K et al. *JAMA Network Open*.

Blinding method
I^2 value for double blind studies is 39.18%
I^2 value for unblinded/single blind studies is 72.04%

Infusion method
I^2 value for the studies including the bolus followed by continuous infusion method is 70.32%
I^2 value for the studies including the continuous infusion alone method is 0%

Single-day versus multiple-day studies
I^2 value for single-day studies is 64.59%
I^2 value for multiple-day studies is 62.56%

©2020 Beck K et al. *JAMA Network Open*.

eMethods 10. Subanalyses of Type of Symptom Scale Used

Total psychopathology
The use of both BPRS and PANSS resulted in a statistically significant effect of ketamine on the positive symptoms. (SMD= 1.45, 95% CI, 1.15 to 1.74, $p < 0.001$, and 1.64, 95% CI, 1.05 to 2.24, $p < 0.001$ respectively). However, there was no significant difference in the magnitude of the association between the two methods ($p=0.56$).

Positive psychotic symptoms
The use of both BPRS and PANSS resulted in a statistically significant effect of ketamine on the positive symptoms. (SMD= 1.68, 95% CI, 0.99 to 2.37, $p < 0.001$, and 1.52, 95% CI, 1.23 to 1.81, $p < 0.001$ respectively). However, there was no significant difference in the magnitude of the association between the two methods ($p=0.67$).

Negative psychotic symptoms
The use of both BPRS and PANSS resulted in a statistically significant effect of ketamine on the positive symptoms. (SMD= 1.27, 95% CI, 0.94, to 1.61, $p < 0.001$, and 1.09, 95% CI, 0.85 to 1.33, $p < 0.001$ respectively). However, there was no significant difference in the magnitude of the association between the two methods ($p=0.38$).

©2020 Beck K et al. *JAMA Network Open*.

eFigure 1. Funnel Plot for Total Symptoms

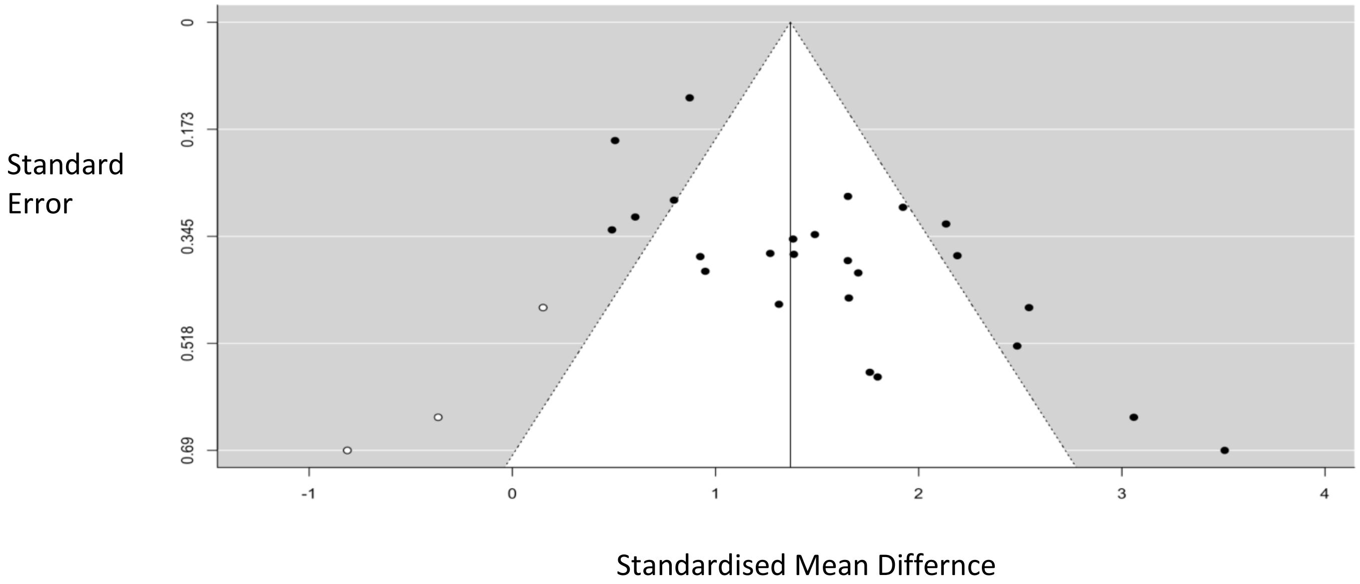

©2020 Beck K et al. *JAMA Network Open*.

eFigure 2. Funnel Plot for Positive Symptoms

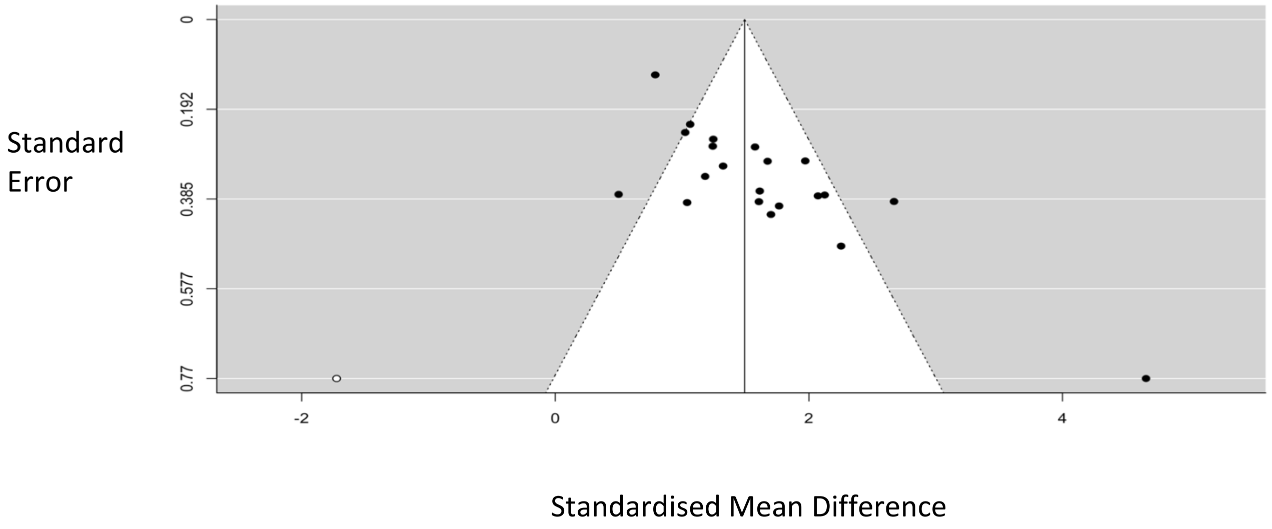

©2020 Beck K et al. *JAMA Network Open*.

eFigure 3. Funnel Plot for Negative Symptoms

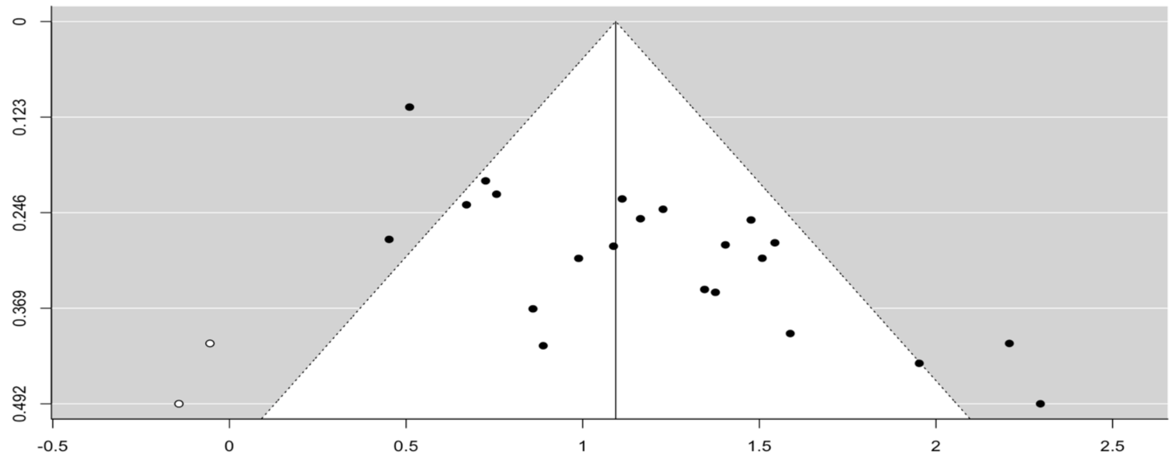

Standardised Mean Difference

©2020 Beck K et al. *JAMA Network Open*.

eFigure 4. Subgroup Analysis of Single-Day vs Multiple-Day Studies for Total Symptoms

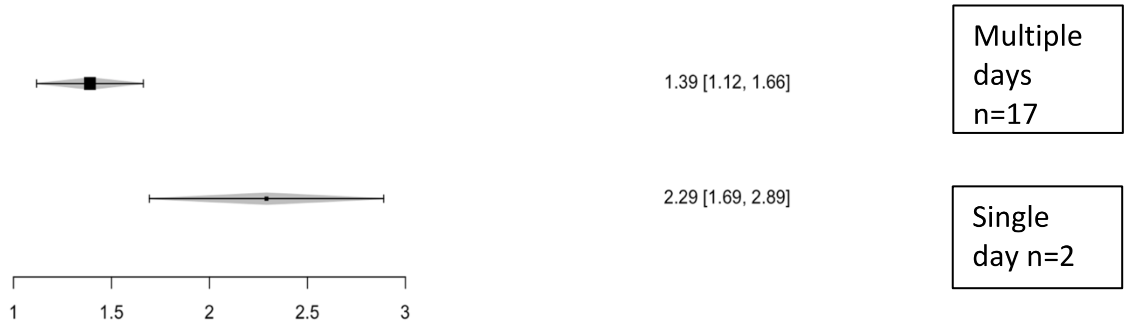

©2020 Beck K et al. *JAMA Network Open*.

eFigure 5. Subgroup Analysis of Method of Infusion (Bolus and a Continuous Infusion vs Only a Continuous Infusion) Positive Symptoms

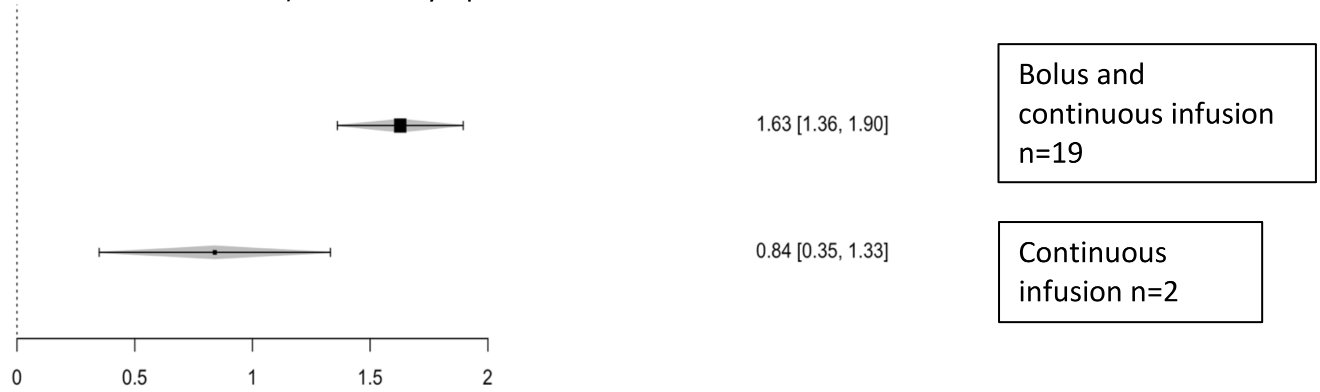

©2020 Beck K et al. *JAMA Network Open*.

eTable 1. Raw Data Used in Total BPRS and PANSS Analysis for Healthy Participants

Author	Year	Sample size	Ketamine mean	Ketamine S.D	Placebo mean	Placebo S.D
**Kraguljac**	2017	15	32.73	4.94	20.6	0.74
**Kort**	2017	31	20.52	5.81	8.06	0.25
**Parwani**	2005	13	27.8	7.3	18.7	1.4
**Rowland**	2005	10	25.4	8.2	21	8.2
**Abel**	2003	8	12.1	5.9	1.1	1.1
**Van Berckel**	1998	18	20.6	1.7	18.2	0.4
**Malhotra**	1997	16	27.1	6.4	18.7	1.6
**Duncan**	2001	16	26.4	5.1	18	0.6
**Rowland**	2010	9	23.8	3.7	20.1	0.4
**Newcomer**	1999	15	23.36	2.68	19.17	1.5
**Stone**	2011	8	9.6	4.7	0.9	0.7
**Passie**	2003	12	33	5.32	23.08	5.79
**Hoflich**	2015	30	54.1	14.6	31.1	2.4
**Nagels**	2011	15	53.27	6.71	30.53	1.4
**Vernaleken**	2013	10	46.8	16.6	30	0
**Horacek**	2010	20	38.35	14.82	0.65	1.26
**Krystal**	2006	31	40.2	21.21	29.2	0
**Grent-‘t-Jong**	2018	14	60.1	8.6	35.7	3.37
**Mathalon**	2014	9	45.7	10.8	30	0
**Abdallah**	2018	14	32.71	11.94	17.43	5.095
**D'Souza**	2012	32	57.97	15.11	30.56	2.84
**Morgan**	2011	16	11.06	4.89	7.06	0.25
**Boeijinga**	2007	12	23	6.92	19	2.08
**Thiebes**	2017	24	53.93	10.72	31.04	1.37
**Dickerson**	2010	93	35.2	5.75	30.47	0.85

©2020 Beck K et al. *JAMA Network Open*.

eTable 2. Raw Data Used in Positive BPRS and PANSS Analysis for Healthy Participants

Author	Year	Sample Size	Ketamine mean	Ketamine S.D	Placebo mean	Placebo S.D
**Kraguljac**	2017	15	5.87	1.69	3	0
**Anand**	2000	16	8.5	2.42	4	0
**Newcomer**	1999	15	5	1.6	3	0
**Krystal**	1998	23	9.2	2.55	4.2	0.74
**Breier**	1997	17	9.2	3.2	4.4	1
**Krystal**	1999	20	7.3	0.68	4	0
**Krystal**	2003	26	8	3.88	3.9	0
**Micallef**	2002	8	6.7	4.8	4	0
**Thiebes**	2017	24	11.54	3.86	6.96	0.69
**Powers**	2015	19	12.8	4.14	7.08	0
**Hoflich**	2015	30	14.5	4.7	7.2	0.4
**Nagels**	2011	15	12.5	2.22	7.3	2.14
**Vernaleken**	2013	10	10.3	2.9	7	0
**Krystal**	2005	27	9.04	2.14	6.65	0.89
**Krystal**	2006	31	9.8	3.14	6.8	1.16
**Kleinloog**	2015	30	13	2.5	6.8	0.6
**Grent-‘t-Jong**	2018	14	7.2	1.87	4.1	0.37
**D'Souza**	2018	26	10.35	2.04	7.15	0.46
**Driesen**	2013	22	11.18	2.06	7.18	0.5
**D'Souza**	2012	32	16.25	4.69	7.47	0.8
**Dickerson**	2010	93	8.87	2.27	7.22	0.47

©2020 Beck K et al. *JAMA Network Open*.

eTable 3. Raw Data Used in Negative BPRS and PANSS Analysis for Healthy Participants

Author	Year	Sample Size	Ketamine mean	Ketamine S.D	Placebo mean	Placebo S.D
**Kraguljac**	2017	15	6.87	1.96	3.13	0.35
**Anand**	2000	16	10.5	5.3	3.4	0.94
**Krystal**	1998	23	7.3	2.83	3.4	0.94
**Krystal**	1999	20	7.8	2	3.2	0
**Krystal**	2003	26	6.8	3	3.2	0
**Duncan**	2001	16	5.8	3	2.9	0.5
**Micallef**	2002	8	13.1	10.1	3	0
**Malhotra**	1997	16	6.5	2.4	3.5	0.8
**Thiebes**	2017	24	14.33	4.43	8.13	0.34
**Powers**	2015	19	9.77	2.6	7.21	1.18
**Hoflich**	2015	30	9.2	2.9	7.3	1
**Nagels**	2011	15	18.12	5	7.27	1.4
**Vernaleken**	2013	10	15	8.51	7	0
**Krystal**	2005	27	11.61	2.87	7.45	0.62
**Krystal**	2006	31	11.7	4.14	7.52	1.31
**Kleinloog**	2015	30	12	3.8	7.7	1
**Grent-‘t-Jong**	2018	14	13.5	3.74	8	2.24
**Abdallah**	2018	14	3.93	2.67	2.79	0.8
**D'Souza**	2018	26	8.54	1.27	7.65	0.94
**Driesen**	2013	22	8.68	2.1	7.41	0.91

©2020 Beck K et al. *JAMA Network Open*.

**D'Souza**	2012	32	14.38	5.33	7.06	1.22
**Dickerson**	2010	93	7.87	1.41	7.24	0.6

©2020 Beck K et al. *JAMA Network Open*.

eTable 4. Raw Data used in Total, Negative and Positive BPRS and PANSS Analysis for People With Schizophrenia

	n	**Placebo condition**<br>Total: Positive: Negative<br>Mean (S.D)	**Ketamine Condition**<br>Total: Positive: Negative<br>Mean (S.D)
Malhotra 1997[59]	13	31.9 (8.7)	40.1 (10.5)
Malhotra 1998[65]	18	NR: 8.6 (2.7): 7.6 (2.9)	NR:13.1 (4.0): 9.0 (4.1)
Lahti 2001 [20]	17	-0.9 (-3): 0.1 (0.8): 0.2 (0.8)	6.8 (-9): 3.3 (2.4): 1 (3.1)

NR: Not reported

©2020 Beck K et al. *JAMA Network Open*.

eTable 5. Study Description, Ketamine Method, Placebo Condition, Symptoms (BPRS and PANSS), and Exclusion Criteria in Studies Examining Acute Ketamine Administration in Healthy Controls

Study	Blinded	Randomised	Ketamine method of administration/ dose/timing	Placebo condition	Symptoms (BPRS)	Exclusion Criteria
**Kraguljac 2017** [66]	No	No	IV bolus of ketamine 0.27mg/kg over 10mins. Followed by continuous infusion (0.25mg/kg/h, flow rate of 0.01 ml/s). No information on length of infusion<br><br>Time between placebo and ketamine test: same day	saline	Total<br>Positive (2)<br>Negative	Family history of psychosis, major medical or neurological disorder, prior exposure to ketamine, concurrent psychotropic medication use
**Kort 2017** [49]	Double	Yes	IV bolus of ketamine 0.23mg/kg over 1min. Followed by 0.58 mg/kg/hour for 30mins. Followed by 0.29 mg/kg/hour for 50 mins.<br><br>Time between placebo and ketamine test: 12.65+/-11.92 days<br><br>Fasted prior to ketamine: yes	saline	Total	Past or present psychiatric history, recent substance misuse, family history of psychosis, major medical or neurological disorder, concurrent psychotropic medication use
**Duncan 2001** [50]	Double	Yes	IV ketamine 0.5mg/kg infused over 60mins<br><br>Time between placebo and ketamine test: 8 days (range 4-21)<br><br>Time of day ketamine given: morning<br><br>Fasted prior to ketamine: yes	saline	Total<br>Negative<br><br>Timings: 50mins	Past or present psychiatric history, substance dependence, family history of psychosis, major medical or neurological disorder
**Parwani 2005** [51]	Double	Yes	IV bolus ketamine 0.27 mg/kg over 10mins. Followed by 0.12 mg/kg over the remaining 50 mins<br><br>Time between placebo and ketamine test: 11 days(range 1-75)<br><br>Time of day ketamine given: morning<br><br>Fasted prior to ketamine: yes	saline	Total<br><br>Timing: 15 mins after infusion start	Past or present psychiatric history, recent substance misuse, major medical or neurological disorder

©2020 Beck K et al. *JAMA Network Open*.

**Rowland 2005** [67]	Double	Yes	IV bolus of ketamine 0.27mg/kg over 10mins. Followed by 0.00225 mg/kg per min for up to 120mins<br><br>Time between placebo and ketamine test: 7-14 days	saline	Total<br><br>Timing: 45 mins after infusion start	Past or present psychiatric history, recent substance misuse, family history of psychosis,<br>major medical or neurological disorder
**Abel 2003** [52]	Double	Yes	IV bolus of ketamine 0.23 mg/kg over 5mins. Followed by an infusion of 0.5mg/kg for 40 to 60 mins<br><br>Time between placebo and ketamine test: >7 days	saline	Total<br><br>Timing: 15 mins after infusion start	Past or present psychiatric history, recent substance misuse, major medical or neurological disorder, prior exposure to ketamine, concurrent psychotropic medication use
**Anand 2000**[68]	Double	Yes	IV bolus of ketamine 0.26 mg/kg for 1 min. Followed by 0.65 mg/kg for 90mins infusion<br><br>Time between placebo and ketamine test: 3-7 days	saline	Positive (1)<br>Negative<br><br>Timing: 5mins after infusion start	Past or present psychiatric history, recent substance misuse, family history of psychosis,<br>major medical or neurological disorder,<br>concurrent psychotropic medication use
**Krystal 1998** [69]	Double	Yes	IV bolus of ketamine 0.26mg/kg for 1 min. Followed IV infusion of 0.65 mg/kg for 60 mins<br><br>Time between placebo and ketamine test: 3-7 days	saline	Positive (1)<br>Negative<br><br>Timing: positive symptoms 60 mins & negative symptoms 60 mins after infusion start	Past or present psychiatric history, recent substance misuse, family history of psychosis,<br>major medical or neurological disorder
**Breier 1997** [70]	Double	Yes	IV bolus of ketamine 0.12 mg/kg. Followed by 0.65mg/kg constant infusion for 60mins<br><br>Time between placebo and ketamine test: 7.8 (6.5) days	saline	Positive (1)	Past or present psychiatric history, recent substance misuse, major medical or neurological disorder

©2020 Beck K et al. *JAMA Network Open*.

**van Berckel 1998** [57]	Double	Yes	IV ketamine 30ml/hr for 40 mins. Followed by 20ml/hr for 10mins. Followed by 10ml/h for 85 mins<br><br>Time between placebo and ketamine test: > 7 days<br><br>Time of day ketamine given: morning<br><br>Fasted prior to ketamine: yes		Total<br><br>Timing: 40mins after infusion start	Past or present psychiatric history, recent substance misuse, family history of psychosis, major medical or neurological disorder
**Malhotra 1997** [19]	Double	Yes	IV ketamine bolus of 0.12mg/kg. Followed by 0.65mg/kg of ketamine (max dose 58 mg) over one hour for a total dose of 0.77mg/kg/hr<br><br>Time between placebo and ketamine test: >1 day<br><br>Time of day ketamine given: morning<br><br>Fasted prior to ketamine: yes	saline	Total<br>Negative<br><br>Timing: 55 mins after infusion start.	Past or present psychiatric history, recent substance misuse, major medical or neurological disorder
**Krystal 1999** [71]	Double	Yes	IV bolus of ketamine 0.26 mg/kg over 1 min. Followed infusion of 0.65 mg/kg for 60mins<br><br>Time between placebo and ketamine test: 3-7 days	saline	Positive (1)<br>Negative<br><br>Timing: Positive symptoms 60mins & negative symptoms 60 mins post infusion start	Past or present psychiatric history, recent substance misuse, family history of psychosis, major medical or neurological disorder, concurrent psychotropic medication use

©2020 Beck K et al. *JAMA Network Open*.

**Krystal 2003** [72]	Double	Yes	IV infusion of 0.5mg/kg ketamine for 40 mins<br><br>Time between placebo and ketamine test: 3-7 days<br><br>Time of day ketamine given: morning<br><br>Fasted prior to ketamine: yes	saline	Positive (1)<br>Negative<br><br>Timing: 80 mins after start of infusion	Past or present psychiatric history, recent substance misuse, family history of psychosis,<br>major medical or neurological disorder,<br>concurrent psychotropic medication use
**Micallef 2002** [73]	Double	Yes	IV ketamine 0.5 mg/kg infusion for 60 mins<br><br>Time between placebo and ketamine test: 7 days	saline	Positive (1)<br>Negative	Past or present psychiatric history, recent substance misuse, family history of psychosis,<br>major medical or neurological disorder,<br>concurrent psychotropic medication use
**Rowland 2010** [53]	Double	Yes	IV bolus of ketamine 0.2mg/kg over 10mins. Followed by maintenance dose of 0.4mg/kg/hr. No information on length.<br><br>Time between placebo and ketamine test: >1 day<br><br>Fasted prior to ketamine: yes	saline	Total	Past or present psychiatric history, recent substance misuse, family history of psychosis,<br>major medical or neurological disorder, prior exposure to ketamine
**Newcomer 1999** [74]	Double	Yes	IV bolus of 0.27 mg/kg of ketamine over 10mins. Followed by infusion of 0.00225 mg/kg/min. No information on the length of the infusion<br><br>Time between placebo and ketamine test: 2.6 +/-1.1 days<br><br>Time of day ketamine given: morning<br><br>Fasted prior to ketamine: yes	saline	Total<br>Positive (1)<br><br>Timings: 30 mins after start of infusion	Past or present psychiatric history, recent substance misuse,<br>major medical or neurological disorder, prior exposure to ketamine

©2020 Beck K et al. *JAMA Network Open*.

**Stone 2011**[75]	Double	Yes	IV bolus of ketamine at a rate of 0.23 mg/kg over 30s followed by an infusion of 0.65 mg/kg/h<br><br>Time between placebo and ketamine test: >1 day	saline	Total	Major medical or neurological disorder, prior exposure to ketamine
**Boeijinga 2007**[28]	Double	Yes	IV bolus of ketamine at a rate of 0.081 mg/kg over 10 mins followed by 0.000675 infusion for 120 mins.<br><br>Time between placebo and ketamine test: 7 days<br><br>Fasted prior to ketamine: yes	saline	Total<br><br>Timings: 30 mins after start of infusion	Past or present psychiatric history, recent substance misuse, family history of psychosis, major medical or neurological disorder, concurrent psychotropic medication use
**Passie 2003** [54]	Double	Yes	IV S-ketamine bolus 5mg over 5 mins. Followed by continuous infusion of 0.005mg/min/kg<br><br>Time between placebo and ketamine test: >7 days<br><br>Time of day ketamine given: afternoon	saline	Total	Past or present psychiatric history, recent substance misuse, major medical or neurological disorder

©2020 Beck K et al. *JAMA Network Open*.

**Abdallah 2018** [76]	Single	No	IV ketamine bolus 0.23 mg/kg. Followed by continuous infusion of 0.58mg/kg over 75 mins.<br><br>Time between placebo and ketamine test: >7 days<br><br>Time of day ketamine given: afternoon	saline	Total<br>Negative<br><br>Timings: 120 mins after start of infusion	Past or present psychiatric history, recent substance abuse, family history of psychosis,<br>major medical or neurological disorder
**Morgan 2011**[77]	Double	no	IV ketamine with targeted plasma concentration of 100 ng/ml for 60 mins and then 200 ng/ml for further 60mins	saline	Total	Past or present psychiatric history, recent substance misuse
**Horacek 2010** [55]	Double	Yes	IV bolus of 0.27 mg/kg of ketamine over 10mins. Followed by infusion of 0.27mg/kg for 20mins.<br><br>Time between placebo and ketamine test: > 14 days	saline	Total<br><br>Timing: 30mins after start of infusion	Past or present psychiatric history, recent substance misuse, family history of psychosis
**Thiebes 2017**[78]	Single	Yes	IV bolus of 10 mg of s-ketamine over 5 mins. Followed by infusion of 0.006 mg/kg/min. As ketamine plasma levels slowly increase with continuous infusion, the dosage was reduced by 10% every 10 minutes, for 75 mins.<br><br>Time between placebo and ketamine test: > 7 days	saline	Total<br>Positive<br>Negative	Past or present psychiatric history, recent substance misuse, family history of psychosis,<br>major medical or neurological disorder

©2020 Beck K et al. *JAMA Network Open*.

**Powers 2015**[79]	No	No	IV bolus of 0.23 mg/kg of ketamine over 1 min. Followed by infusion of 0.58mg/kg/h for 75 mins.	saline	Positive<br>Negative	Past or present psychiatric history, substance dependence, family history of psychosis,<br>major medical or neurological disorder
**Mathalon 2014** [80]	Double	Yes	IV bolus of 0.26 mg/kg of ketamine over 1 min. Followed by infusion of 0.65mg/kg/h for 120 mins.<br><br>Time between placebo and ketamine test: >3 days<br><br>Fasted prior to ketamine: yes	saline	Total<br><br>Timing: 1min	Past or present psychiatric history, substance misuse, family history of psychosis,<br>major medical or neurological disorder
**Hoflich 2015**[81]	Double	Yes	IV bolus of 0.11 mg/kg of s-ketamine over 1 min. Followed by infusion of 0.12 mg/kg for 19 mins.<br><br>Fasted prior to ketamine: yes	saline	Total<br>Positive<br>Negative	Past or present psychiatric history, substance misuse, family history of psychosis,<br>major medical or neurological disorder,<br>concurrent psychotropic medication use
**Nagels 2011**[82]	Double	Yes	IV bolus of 8mg mg/kg of s-ketamine over 5 min. Followed by infusion of 0.01 mg/kg/min for 60 mins.	saline	Total<br>Positive<br>Negative	Past or present psychiatric history, substance misuse, family history of psychosis,<br>major medical or neurological disorder
**Driesen 2013** [83]	No	No	IV bolus of 0.23mg/kg of ketamine over 1 min. Followed by infusion of 0.58mg/kg/min for 45 mins.<br><br>Time between placebo and ketamine test: <1 day	saline	Positive<br>Negative<br><br>Timing 45mins	Past or present psychiatric history, family history of psychosis,<br>major medical or neurological disorder
**Vernaleken 2013** [84]	Single	Yes	IV bolus of 8mg of s-ketamine over 5 min. Followed by infusion of 0.01 mg/kg/min for 60 mins.<br><br>Time between placebo and ketamine test: > 7 days	saline	Total<br>Positive<br>Negative	Past or present psychiatric history, recent substance misuse, major medical or neurological disorder,<br>concurrent psychotropic medication use

©2020 Beck K et al. *JAMA Network Open*.

**Krystal 2005** [85]	Double	Yes	IV bolus of 0.23mg/kg of ketamine over 1 min. Followed by infusion of 0.5 mg/kg for 60 mins.<br><br>Time between placebo and ketamine test: >1 day	saline	Positive<br>Negative<br><br>Timing:<br>Positive symptoms 60 mins & negative symptoms 60 mins.	Past or present psychiatric history, recent substance misuse, family history of psychosis,<br>major medical or neurological disorder,<br>concurrent psychotropic medication use
**Krystal 2006** [86]	Double	Yes	IV bolus of 0.23mg/kg of ketamine over 1 min. Followed by infusion of 0.58 mg/kg/h for 60 mins.<br><br>Time between placebo and ketamine test: > 1 day<br><br>Time of day ketamine given: morning<br><br>Fasted prior to ketamine: yes	saline	Total<br>Positive<br>Negative<br><br>Timing:<br>Total symptoms 80 mins<br>Positive symptoms 80 mins & negative symptoms 10 mins.	Past or present psychiatric history, recent substance misuse, major medical or neurological disorder
**Kleinloog 2015** [87]	Double	Yes	IV s-ketamine with targeted plasma concentration of 240 ng/ml (bolus +continuous design used)<br><br>Time between placebo and ketamine test: > 1 day	saline	Total<br>Positive<br>Negative	Past or present psychiatric history, substance misuse, family history of psychosis,<br>major medical or neurological disorder,<br>concurrent psychotropic medication use
**D'Souza 2012**[88]	Double	Yes	IV bolus of 0.23mg/kg of ketamine over 1 min. Followed by infusion of 0.58mg/kg for 30 mins. Followed by 0.29 mg/kg/hour for 64 mins.<br><br>Time between placebo and ketamine test: >2 days<br><br>Fasted prior to ketamine: yes	saline	Total<br>Positive<br>Negative	Past or present psychiatric history, recent substance misuse, family history of psychosis,<br>major medical or neurological disorder,<br>concurrent psychotropic medication use

©2020 Beck K et al. *JAMA Network Open*.

**Grent-'t-Jong 2018** [89]	Single	Yes	IV bolus of 10mg of s-ketamine. Followed by infusion of 0.006 mg/kg/min for 45 mins.	saline	Total<br>Negative<br>Positive	Major medical or neurological disorder
**D'Souza 2018** [90]	NR	No	IV bolus of 0.23 mg/kg of ketamine over 1 min. Followed by infusion of 0.58 mg/kg/hour for 45 mins.<br><br>Time between placebo and ketamine test: <1 day	saline	Total<br>Negative<br>Positive	Past or present psychiatric history, recent substance misuse, family history of psychosis,<br>major medical or neurological disorder
**Dickerson 2010** [91]	Single	Yes	IV bolus of 0.23 mg/kg of ketamine over 1 min. Followed by infusion of 0.58 mg/kg/hour for 45 mins.<br><br>Time between placebo and ketamine test: >3 days<br><br>Fasted prior to ketamine: yes	saline	Total<br>Negative<br>Positive	Past or present psychiatric history, recent substance misuse, major medical or neurological disorder

BPRS Positive symptoms (1): conceptual disorganisation, suspiciousness, hallucinatory behaviour, unusual thought content
BPRS Positive symptoms (2): conceptual disorganisation, hallucinatory behaviour, unusual thought content
BPRS Negative symptoms: Blunted affect, emotional withdrawal, motor retardation

©2020 Beck K et al. *JAMA Network Open*.

eTable 6. Study Description, Ketamine Administration Method, Placebo Condition, Symptoms (BPRS), and Exclusion Criteria in Studies Examining Acute Ketamine Administration to Patients With Schizophrenia

Study	M:F	Diagnosis	Medication	Blinding	Randomised	Ketamine method of administration/ dose/timing	Placebo condition	Symptoms (BPRS)	Exclusion
Malhotra 1998 [92]	13:5	Schizophrenia Schizoaffective disorder	Fluphenazine (3)<br><br>Clozapine (4)<br><br>Antipsychotic free (11)	Double	yes	IV bolus of 0.12mg/kg of ketamine. Followed by 0.65mg/kg infusion over 60 mins.<br><br>Time between placebo and ketamine test: >1 day		Positive (1) Negative<br><br>Timing: 35 mins after start of infusion	Medical illness, current alcohol or drug abuse
Malhotra 1997 [59]	10:3	Schizophrenia Schizoaffective disorder	Unmedicated for two weeks (12)<br><br>Medication naïve (1)	Double	yes	IV bolus of 0.12mg/kg of ketamine. Followed by 0.65mg/kg of ketamine (max dose 58 mg) over 60mins for a total dose of 0.77mg/kg/hr<br><br>Time between placebo and ketamine test: >1 day<br><br>Time of day ketamine given: morning<br><br>Fasted prior to ketamine: yes	saline	Total<br><br>Timing: 55 mins	Medical illness, current alcohol or drug abuse
Lahti 2001 [20]	11:6	Schizophrenia	Haloperidol 0.3mg/kg/day	Double	yes	IV bolus of 0.3mg/kg of ketamine for 60 secs<br><br>Time between placebo and ketamine test: >1 day		Total Positive (2) Negative<br><br>Timing: 20 mins after infusion start.	Medical illness, no current alcohol or drug abuse

Positive symptoms (1): conceptual disorganisation, suspiciousness, hallucinatory behaviour, unusual thought content
Positive symptoms (2): conceptual disorganisation, hallucinatory behaviour, unusual thought content
Negative symptoms: Blunted affect, emotional withdrawal, motor retardation

©2020 Beck K et al. *JAMA Network Open*.

eReferences.

1. Krystal JH, Bennett A, Abi-Saab D, et al. Dissociation of ketamine effects on rule acquisition and rule implementation: possible relevance to NMDA receptor contributions to executive cognitive functions. *Biol Psychiatry*. 2000;47(2):137-143.
2. Abel KM, Allin MPG, Kucharska-Pietura K, et al. Ketamine alters neural processing of facial emotion recognition in healthy men: an fMRI study. *Neuroreport*. 2003;14(3):387-391.
3. Umbricht D, Koller R, Vollenweider FX, Schmid L. Mismatch negativity predicts psychotic experiences induced by NMDA receptor antagonist in healthy volunteers. *Biol Psychiatry*. 2002;51(5):400-406.
4. Malhotra AK, Pinals DA, Weingartner H, et al. NMDA receptor function and human cognition: the effects of ketamine in healthy volunteers. *Neuropsychopharmacology*. 1996;14(5):301-307.
5. Krystal JH, Karper LP, Seibyl JP, et al. Subanesthetic effects of the noncompetitive NMDA antagonist, ketamine, in humans. Psychotomimetic, perceptual, cognitive, and neuroendocrine responses. *Arch Gen Psychiatry*. 1994;51(3):199-214.
6. Passie T, Karst M, Wiese B, Emrich HM, Schneider U. Effects of different subanesthetic doses of (S)-ketamine on neuropsychology, psychopathology, and state of consciousness in man. *Neuropsychobiology*. 2005;51(4):226-233. doi:10.1159/000085724
7. Rowland LM, Bustillo JR, Mullins PG, et al. Effects of Ketamine on Anterior Cingulate Glutamate Metabolism in Healthy Humans: A 4-T Proton MRS Study. *Am J Psychiatry*. 2005;162(2):394-396. doi:10.1176/appi.ajp.162.2.394
8. Rivolta D, Heidegger T, Scheller B, et al. Ketamine Dysregulates the Amplitude and Connectivity of High-Frequency Oscillations in Cortical-Subcortical Networks in Humans: Evidence From Resting-State Magnetoencephalography-Recordings. *Schizophr Bull*. 2015;41(5):1105-1114. doi:https://dx.doi.org/10.1093/schbul/sbv051
9. Mueller F, Musso F, London M, de Boer P, Zacharias N, Winterer G. Pharmacological fMRI: Effects of subanesthetic ketamine on resting-state functional connectivity in the default mode network, salience network, dorsal attention network and executive control network. *NeuroImage Clin*. 2018;19:745-757. doi:https://dx.doi.org/10.1016/j.nicl.2018.05.037
10. Nagels A, Cabanis M, Oppel A, Kirner-Veselinovic A, Schales C, Kircher T. S-Ketamine-Induced NMDA Receptor Blockade during Natural Speech Production and Its Implications for Formal Thought Disorder in Schizophrenia: A Pharmaco-fMRI Study. *Neuropsychopharmacology*. 2018;43(6):1324-1333. doi:https://dx.doi.org/10.1038/npp.2017.270
11. Driesen NR, McCarthy G, Bhagwagar Z, et al. The impact of NMDA receptor blockade on human working memory-related prefrontal function and connectivity. *Neuropsychopharmacology*. 2013;38(13):2613-2622. doi:https://dx.doi.org/10.1038/npp.2013.170
12. Thiebes S, Curic S, Polomac N, et al. The effects of glutamatergic modulation on human auditory perception and behavioural changes in a Mismatch Negativity (MMN) paradigm. *Pharmacopsychiatry*. 2015;48(06):A60. doi:10.1055/s-0035-1557998
13. Oranje B, van Berckel BN, Kemner C, van Ree JM, Kahn RS, Verbaten MN. The effects

of a sub-anaesthetic dose of ketamine on human selective attention. *Neuropsychopharmacology*. 2000;22(3):293-302.

14. Curic S, Leicht G, Thiebes S, et al. Reduced auditory evoked gamma-band response and schizophrenia-like clinical symptoms under subanesthetic ketamine. *Neuropsychopharmacology*. 2019. doi:https://dx.doi.org/10.1038/s41386-019-0328-5
15. Weiler MA, Thaker GK, Lahti AC, Tamminga CA. Ketamine effects on eye movements. *Neuropsychopharmacology*. 2000;23(6):645-653.
16. Nagels A, Kirner-Veselinovic A, Wiese R, Paulus FM, Kircher T, Krach S. Effects of ketamine-induced psychopathological symptoms on continuous overt rhyme fluency. *Eur Arch Psychiatry Clin Neurosci*. 2012;262(5):403-414. doi:https://dx.doi.org/10.1007/s00406-011-0281-8
17. Thiebes S, Steinmann S, Curic S, et al. Alterations in interhemispheric gamma-band connectivity are related to the emergence of auditory verbal hallucinations in healthy subjects during NMDA-receptor blockade. *Neuropsychopharmacology*. 2018;43(7):1608-1615. doi:https://dx.doi.org/10.1038/s41386-018-0014-z
18. Medoff DR, Holcomb HH, Lahti AC, Tamminga CA. Probing the human hippocampus using rCBF: Contrasts in schizophrenia. *Hippocampus*. 2001;11(5):543-550. doi:10.1002/hipo.1070
19. Malhotra AK, Pinals DA, Adler CM, et al. Ketamine-induced exacerbation of psychotic symptoms and cognitive impairment in neuroleptic-free schizophrenics. *Neuropsychopharmacology*. 1997;17(3):141-150.
20. Lahti AC, Weiler MA, Tamara Michaelidis B., Parwani A, Tamminga CA. Effects of Ketamine in Normal and Schizophrenic Volunteers. *Neuropsychopharmacology*. 2001;25(4):455-467. doi:10.1016/S0893-133X(01)00243-3
21. Avila MT, Weiler MA, Lahti AC, Tamminga CA, Thaker GK. Effects of ketamine on leading saccades during smooth-pursuit eye movements may implicate cerebellar dysfunction in schizophrenia. *Am J Psychiatry*. 2002;159(9):1490-1496.
22. Kraguljac NV, Carle M, Frolich MA, et al. Mnemonic Discrimination Deficits in First-Episode Psychosis and a Ketamine Model Suggests Dentate Gyrus Pathology Linked to N-Methyl-D-Aspartate Receptor Hypofunction. *Biol psychiatry Cogn Neurosci neuroimaging*. 2018;3(3):231-238. doi:https://dx.doi.org/10.1016/j.bpsc.2017.02.005
23. Holcomb HH, Lahti AC, Medoff DR, Weiler M, Tamminga CA. Sequential regional cerebral blood flow brain scans using PET with H2(15)O demonstrate ketamine actions in CNS dynamically. *Neuropsychopharmacology*. 2001;25(2):165-172.
24. LaPorte DJ, Blaxton TA, Michaelidis T, et al. Subtle effects of ketamine on memory when administered following stimulus presentation. *Psychopharmacology (Berl)*. 2005;180(3):385-390.
25. Kegeles LS, Abi-Dargham A, Zea-Ponce Y, et al. Modulation of amphetamine-induced striatal dopamine release by ketamine in humans: implications for schizophrenia. *Biol Psychiatry*. 2000;48(7):627-640.
26. Ahn K-H, Youn T, Cho SS, et al. N-methyl-D-aspartate receptor in working memory impairments in schizophrenia: event-related potential study of late stage of working memory process. *Prog Neuropsychopharmacol Biol Psychiatry*. 2003;27(6):993-999.
27. Kegeles LS, Martinez D, Kochan LD, et al. NMDA antagonist effects on striatal dopamine release: positron emission tomography studies in humans. *Synapse*. 2002;43(1):19-29.
28. Boeijinga PH, Soufflet L, Santoro F, Luthringer R. Ketamine effects on CNS responses

assessed with MEG/EEG in a passive auditory sensory-gating paradigm: an attempt for modelling some symptoms of psychosis in man. *J Psychopharmacol*. 2007;21(3):321-337. doi:10.1177/0269881107077768
29. Adler CM, Goldberg TE, Malhotra AK, Pickar D, Breier A. Effects of ketamine on thought disorder, working memory, and semantic memory in healthy volunteers. *Biol Psychiatry*. 1998;43(11):811-816. http://ovidsp.ovid.com/ovidweb.cgi?T=JS&PAGE=reference&D=med4&NEWS=N&AN=9611670.
30. Gunduz-Bruce H, Reinhart RMG, Roach BJ, et al. Glutamatergic modulation of auditory information processing in the human brain. *Biol Psychiatry*. 2012;71(11):969-977. doi:https://dx.doi.org/10.1016/j.biopsych.2011.09.031
31. Holcomb HH, Lahti AC, Medoff DR, Cullen T, Tamminga CA. Effects of noncompetitive NMDA receptor blockade on anterior cingulate cerebral blood flow in volunteers with schizophrenia. *Neuropsychopharmacology*. 2005;30(12):2275-2282.
32. Vinckier F, Gaillard R, Palminteri S, et al. Confidence and psychosis: a neuro-computational account of contingency learning disruption by NMDA blockade. *Mol Psychiatry*. 2016;21(7):946-955. doi:https://dx.doi.org/10.1038/mp.2015.73
33. Oranje B, Gispen-de Wied CC, Westenberg HG, Kemner C, Verbaten MN, Kahn RS. Haloperidol counteracts the ketamine-induced disruption of processing negativity, but not that of the P300 amplitude. *Int J Neuropsychopharmacol*. 2009;12(6):823-832.
34. Moaddel R, Shardell M, Khadeer M, et al. Plasma metabolomic profiling of a ketamine and placebo crossover trial of major depressive disorder and healthy control subjects. Ali-Sisto Anderson, Baranyi, Berman, Bersani, Cervenka, Chrapko, Chrapko, Darlington, Diazgranados, DiazGranados, Dinoff, Dobos, Domin, First, Forstermann, Gowda, Gracia-Garcia, Guldbrandt, Harraz, Heisler, Hess, Iadarola, Kessler, Kiecolt-Glaser, Kornhu A, ed. *Psychopharmacology (Berl)*. 2018;235(10):3017-3030. doi:http://dx.doi.org/10.1007/s00213-018-4992-7
35. Musso F, Brinkmeyer J, Ecker D, et al. Ketamine effects on brain function-simultaneous fMRI/EEG during a visual oddball task. *Neuroimage*. 2011;58(2):508-525. doi:https://dx.doi.org/10.1016/j.neuroimage.2011.06.045
36. Honey GD, Corlett PR, Absalom AR, et al. Individual differences in psychotic effects of ketamine are predicted by brain function measured under placebo. *J Neurosci*. 2008;28(25):6295-6303. doi:https://dx.doi.org/10.1523/JNEUROSCI.0910-08.2008
37. Aalto S, Hirvonen J, Kajander J, et al. Ketamine does not decrease striatal dopamine D2 receptor binding in man. *Psychopharmacology (Berl)*. 2002;164(4):401-406.
38. Aalto S, Ihalainen J, Hirvonen J, et al. Cortical glutamate-dopamine interaction and ketamine-induced psychotic symptoms in man. *Psychopharmacology (Berl)*. 2005;182(3):375-383.
39. Abel KM, Allin MP., Hemsley DR, Geyer MA. Low dose ketamine increases prepulse inhibition in healthy men. *Neuropharmacology*. 2003;44(6):729-737. doi:10.1016/S0028-3908(03)00073-X
40. Stone JM, Erlandsson K, Arstad E, et al. Relationship between ketamine-induced psychotic symptoms and NMDA receptor occupancy: a [(123)I]CNS-1261 SPET study. *Psychopharmacology (Berl)*. 2008;197(3):401-408. doi:https://dx.doi.org/10.1007/s00213-007-1047-x
41. Fu CHY, Abel KM, Allin MPG, et al. Effects of ketamine on prefrontal and striatal
©2020 Beck K et al. *JAMA Network Open*.

regions in an overt verbal fluency task: a functional magnetic resonance imaging study. *Psychopharmacology (Berl)*. 2005;183(1):92-102.
42. Dandash O, Harrison BJ, Adapa R, et al. Selective augmentation of striatal functional connectivity following NMDA receptor antagonism: implications for psychosis. *Neuropsychopharmacology*. 2015;40(3):622-631. doi:https://dx.doi.org/10.1038/npp.2014.210
43. Höflich A, Hahn A, Küblböck M, et al. Ketamine-dependent neuronal activation in healthy volunteers. *Brain Struct Funct*. 2017;222(3):1533-1542. doi:10.1007/s00429-016-1291-0
44. Umbricht D, Schmid L, Koller R, Vollenweider FX, Hell D, Javitt DC. Ketamine-Induced Deficits in Auditory and Visual Context-Dependent Processing in Healthy Volunteers. *Arch Gen Psychiatry*. 2000;57(12):1139. doi:10.1001/archpsyc.57.12.1139
45. Krystal JH, Abi-Saab W, Perry E, et al. Preliminary evidence of attenuation of the disruptive effects of the NMDA glutamate receptor antagonist, ketamine, on working memory by pretreatment with the group II metabotropic glutamate receptor agonist, LY354740, in healthy human subjects. *Psychopharmacology (Berl)*. 2005;179(1):303-309. doi:10.1007/s00213-004-1982-8
46. Daumann J, Heekeren K, Neukirch A, Thiel CM, Moller-Hartmann W, Gouzoulis-Mayfrank E. Pharmacological modulation of the neural basis underlying inhibition of return (IOR) in the human 5-HT2A agonist and NMDA antagonist model of psychosis. *Psychopharmacology (Berl)*. 2008;200(4):573-583. doi:https://dx.doi.org/10.1007/s00213-008-1237-1
47. Deakin JFW, Lees J, McKie S, Hallak JEC, Williams SR, Dursun SM. Glutamate and the neural basis of the subjective effects of ketamine: a pharmaco-magnetic resonance imaging study. *Arch Gen Psychiatry*. 2008;65(2):154-164. doi:https://dx.doi.org/10.1001/archgenpsychiatry.2007.37
48. Hong LE, Summerfelt A, Buchanan RW, et al. Gamma and delta neural oscillations and association with clinical symptoms under subanesthetic ketamine. *Neuropsychopharmacology*. 2010;35(3):632-640. doi:https://dx.doi.org/10.1038/npp.2009.168
49. Kort NS, Ford JM, Roach BJ, et al. Role of N-Methyl-D-Aspartate Receptors in Action-Based Predictive Coding Deficits in Schizophrenia. *Biol Psychiatry*. 2017;81(6):514-524. doi:https://dx.doi.org/10.1016/j.biopsych.2016.06.019
50. Duncan EJ, Madonick SH, Parwani A, et al. Clinical and Sensorimotor Gating Effects of Ketamine in Normals. *Neuropsychopharmacology*. 2001;25(1):72-83. doi:10.1016/S0893-133X(00)00240-2
51. Parwani A, Weiler MA, Blaxton TA, et al. The effects of a subanesthetic dose of ketamine on verbal memory in normal volunteers. *Psychopharmacology (Berl)*. 2005;183(3):265-274. doi:10.1007/s00213-005-0177-2
52. Abel KM, Allin MPG, Kucharska-Pietura K, et al. Ketamine and fMRI BOLD signal: Distinguishing between effects mediated by change in blood flow versus change in cognitive state. *Hum Brain Mapp*. 2003;18(2):135-145. doi:10.1002/hbm.10064
53. Rowland LM, Beason-Held L, Tamminga CA, Holcomb HH. The interactive effects of ketamine and nicotine on human cerebral blood flow. *Psychopharmacology (Berl)*. 2010;208(4):575-584. doi:10.1007/s00213-009-1758-2
54. Passie T, Karst M, Borsutzky M, Wiese B, Emrich HM, Schneider U. Effects of different subanaesthetic doses of (S)-ketamine on psychopathology and binocular depth
©2020 Beck K et al. *JAMA Network Open*.

inversion in man. *J Psychopharmacol*. 2003;17(1):51-56. doi:10.1177/0269881103017001698
55. Horacek J, Brunovsky M, Novak T, et al. Subanesthetic dose of ketamine decreases prefrontal theta cordance in healthy volunteers: implications for antidepressant effect. *Psychol Med*. 2010;40(09):1443-1451. doi:10.1017/S0033291709991619
56. Vollenweider FX, Vontobel P, Oye I, Hell D, Leenders KL. Effects of (S)-ketamine on striatal dopamine: a [11C]raclopride PET study of a model psychosis in humans. *J Psychiatr Res*. 2000;34(1):35-43.
57. van Berckel BN, Oranje B, van Ree JM, Verbaten MN, Kahn RS. The effects of low dose ketamine on sensory gating, neuroendocrine secretion and behavior in healthy human subjects. *Psychopharmacology (Berl)*. 1998;137(3):271-281.
58. Malhotra AK, Adler CM, Kennison SD, Elman I, Pickar D, Breier A. Clozapine blunts N-methyl-D-aspartate antagonist-induced psychosis: A study with ketamine. *Biol Psychiatry*. 1997;42(8):664-668.
59. Malhotra AK, Pinals DA, Adler CM, et al. Ketamine-induced exacerbation of psychotic symptoms and cognitive impairment in neuroleptic-free schizophrenics. *Neuropsychopharmacology*. 1997;17(3):141-150.
60. Lahti A, Koffel B, LaPorte D, Tamminga CA. Subanesthetic Doses of Ketamine Stimulate Psychosis in Schizophrenia. *Neuropsychopharmacology*. 1995;13(1):9-19. doi:10.1016/0893-133X(94)00131-I
61. LaPorte DJ, Lahti AC, Koffel B, Tamminga CA. Absence of ketamine effects on memory and other cognitive functions in schizophrenia patients. *J Psychiatr Res*. 1996;30(5):321-330.
62. Lahti AC, Holcomb HH, Medoff DR, Tamminga CA. Ketamine activates psychosis and alters limbic blood flow in schizophrenia. *Neuroreport*. 1995;6(6):869-872.
63. Lahti AC, Warfel D, Michaelidis T, Weiler MA, Frey K, Tamminga CA. Long-term outcome of patients who receive ketamine during research. *Biol Psychiatry*. 2001;49(10):869-875.
64. https://handbook-5-1.cochrane.org/chapter_8/table_8_7_a_possible_approach_for_summary_assessments_of_the.htm, http://handbook-5-1.cochrane.org/).
65. Malhotra A, Breier A, Goldman D, Picken L, Pickar D. The Apolipoprotein E ε4 Allele Is Associated with Blunting of Ketamine-Induced Psychosis in Schizophrenia A Preliminary Report. *Neuropsychopharmacology*. 1998;19(5):445-448. doi:10.1016/S0893-133X(98)00031-1
66. Kraguljac N V, Frölich MA, Tran S, et al. Ketamine modulates hippocampal neurochemistry and functional connectivity: a combined magnetic resonance spectroscopy and resting-state fMRI study in healthy volunteers. *Mol Psychiatry*. 2017;22(4):562-569. doi:10.1038/mp.2016.122
67. Rowland LM, Astur RS, Jung RE, Bustillo JR, Lauriello J, Yeo RA. Selective cognitive impairments associated with NMDA receptor blockade in humans. *Neuropsychopharmacology*. 2005;30(3):633-639.
68. Anand A, Charney DS, Oren DA, et al. Attenuation of the neuropsychatric effects of ketamine with lamotrigine: Support for hyperglutamatergic effects of N-methyl-D-aspartate receptor antagonists. *Arch Gen Psychiatry*. 2000;57(3):270-276. doi:http://dx.doi.org/10.1001/archpsyc.57.3.270
69. Krystal JH, Karper LP, Bennett A, et al. Interactive effects of subanesthetic ketamine
©2020 Beck K et al. *JAMA Network Open*.

and subhypnotic lorazepam in humans. *Psychopharmacology (Berl)*. 1998;135(3):213-229. doi:10.1007/s002130050503
70. Breier A, Malhotra AK, Pinals DA, Weisenfeld NI, Pickar D. Association of ketamine-induced psychosis with focal activation of the prefrontal cortex in healthy volunteers. *Am J Psychiatry*. 1997. doi:10.1176/ajp.154.6.805
71. Krystal JH, D'Souza DC, Karper LP, et al. Interactive effects of subanesthetic ketamine and haloperidol in healthy humans. *Psychopharmacology (Berl)*. 1999;145(2):193-204. doi:10.1007/s002130051049
72. Krystal JH, Petrakis IL, Limoncelli D, et al. Altered NMDA Glutamate Receptor Antagonist Response in Recovering Ethanol-Dependent Patients. *Neuropsychopharmacology*. 2003;28(11):2020-2028. doi:10.1038/sj.npp.1300252
73. Micallef J, Guillermain Y, Tardieu S, et al. Effects of subanesthetic doses of katamine on sensorimotor information processing in health subjects. *Clin Neuropharmacol*. 2002;25(2):101-106. doi:http://dx.doi.org/10.1097/00002826-200203000-00008
74. Newcomer JW, Farber NB, Jevtovic-Todorovic V, et al. Ketamine-induced NMDA receptor hypofunction as a model of memory impairment and psychosis. *Neuropsychopharmacology*. 1999;20(2):106-118. doi:10.1016/S0893-133X(98)00067-0
75. Stone JM, Abel KM, Allen MPG, et al. Ketamine-Induced Disruption of Verbal Self-Monitoring Linked to Superior Temporal Activation. *Pharmacopsychiatry*. 2010;44(01):33-48. doi:10.1055/s-0030-1267942
76. Abdallah CG, De Feyter HM, Averill LA, et al. The effects of ketamine on prefrontal glutamate neurotransmission in healthy and depressed subjects. *Neuropsychopharmacology*. 2018;43(10):2154-2160. doi:https://dx.doi.org/10.1038/s41386-018-0136-3
77. Morgan HL, Turner DC, Corlett PR, et al. Exploring the impact of ketamine on the experience of illusory body ownership. *Biol Psychiatry*. 2011;69(1):35-41. doi:https://dx.doi.org/10.1016/j.biopsych.2010.07.032
78. Thiebes S, Leicht G, Curic S, et al. Glutamatergic deficit and schizophrenia-like negative symptoms: new evidence from ketamine-induced mismatch negativity alterations in healthy male humans. *J Psychiatry Neurosci*. 2017;42(4):273-283.
79. Powers AR 3rd, Gancsos MG, Finn ES, Morgan PT, Corlett PR. Ketamine-Induced Hallucinations. *Psychopathology*. 2015;48(6):376-385. doi:https://dx.doi.org/10.1159/000438675
80. Mathalon DH, Ahn K-H, Perry EBJ, et al. Effects of nicotine on the neurophysiological and behavioral effects of ketamine in humans. *Front psychiatry*. 2014;5:3. doi:https://dx.doi.org/10.3389/fpsyt.2014.00003
81. Hoflich A, Hahn A, Kublbock M, et al. Ketamine-Induced Modulation of the Thalamo-Cortical Network in Healthy Volunteers As a Model for Schizophrenia. *Int J Neuropsychopharmacol*. 2015;18(9). doi:https://dx.doi.org/10.1093/ijnp/pyv040
82. Nagels A, Kirner-Veselinovic A, Krach S, Kircher T. Neural correlates of S-ketamine induced psychosis during overt continuous verbal fluency. *Neuroimage*. 2011;54(2):1307-1314. doi:10.1016/J.NEUROIMAGE.2010.08.021
83. Driesen NR, McCarthy G, Bhagwagar Z, et al. Relationship of resting brain hyperconnectivity and schizophrenia-like symptoms produced by the NMDA receptor antagonist ketamine in humans. *Mol Psychiatry*. 2013;18(11):1199-1204. doi:https://dx.doi.org/10.1038/mp.2012.194

©2020 Beck K et al. *JAMA Network Open*.

84. Vernaleken I, Klomp M, Moeller O, et al. Vulnerability to psychotogenic effects of ketamine is associated with elevated D2/3-receptor availability. *Int J Neuropsychopharmacol*. 2013;16(04):745-754. doi:10.1017/S1461145712000764
85. Krystal JH, Perry EB, Gueorguieva R, et al. Comparative and Interactive Human Psychopharmacologic Effects of Ketamine and Amphetamine. *Arch Gen Psychiatry*. 2005;62(9):985. doi:10.1001/archpsyc.62.9.985
86. Krystal JH, Madonick S, Perry E, et al. Potentiation of low dose ketamine effects by naltrexone: potential implications for the pharmacotherapy of alcoholism. *Neuropsychopharmacology*. 2006;31(8):1793-1800.
87. Kleinloog D, den Boogaard AU, Dahan A, et al. Optimizing the glutamatergic challenge model for psychosis, using S(+)-ketamine to induce psychomimetic symptoms in healthy volunteers. *J Psychopharmacol*. 2015;29(4):401-413. doi:http://dx.doi.org/10.1177/0269881115570082
88. D'Souza DC, Ahn K, Bhakta S, et al. Nicotine fails to attenuate ketamine-induced cognitive deficits and negative and positive symptoms in humans: implications for schizophrenia. *Biol Psychiatry*. 2012;72(9):785-794. doi:https://dx.doi.org/10.1016/j.biopsych.2012.05.009
89. Grent-'t-Jong T, Rivolta D, Gross J, et al. Acute ketamine dysregulates task-related gamma-band oscillations in thalamo-cortical circuits in schizophrenia. *Brain*. 2018;141(8):2511-2526. doi:https://dx.doi.org/10.1093/brain/awy175
90. D'Souza DC, Carson RE, Driesen N, et al. Dose-Related Target Occupancy and Effects on Circuitry, Behavior, and Neuroplasticity of the Glycine Transporter-1 Inhibitor PF-03463275 in Healthy and Schizophrenia Subjects. *Biol Psychiatry*. 2018;84(6):413-421. doi:https://dx.doi.org/10.1016/j.biopsych.2017.12.019
91. Dickerson D, Pittman B, Ralevski E, et al. Ethanol-like effects of thiopental and ketamine in healthy humans. *J Psychopharmacol*. 2010;24(2):203-211. doi:10.1177/0269881108098612
92. Malhotra AK, Breier A, Goldman D, Picken L, Pickar D. The apolipoprotein E epsilon 4 allele is associated with blunting of ketamine-induced psychosis in schizophrenia. A preliminary report. *Neuropsychopharmacology*. 1998;19(5):445-448.

©2020 Beck K et al. *JAMA Network Open*.
